# Different ways to transport ammonia in human and *Mycobacterium tuberculosis* NAD^+^ synthetases

**DOI:** 10.1038/s41467-019-13845-4

**Published:** 2020-01-07

**Authors:** Watchalee Chuenchor, Tzanko I. Doukov, Kai-Ti Chang, Melissa Resto, Chang-Soo Yun, Barbara Gerratana

**Affiliations:** 1 Departments of Chemistry and Biochemistry, University of Maryland, College Park, MD 20742 USA; 2 Stanford Synchrotron Radiation Lightsource, Menlo Park, CA 94025 USA; 3 Therapeutics & Biotechnology Division, Korea Research Institute of Chemical Technology, Daejeon, 34114 Korea

**Keywords:** X-ray crystallography, Tuberculosis

## Abstract

NAD^+^ synthetase is an essential enzyme of de novo and recycling pathways of NAD^+^ biosynthesis in *Mycobacterium tuberculosis* but not in humans. This bifunctional enzyme couples the NAD^+^ synthetase and glutaminase activities through an ammonia tunnel but free ammonia is also a substrate. Here we show that the *Homo sapiens* NAD^+^ synthetase (hsNadE) lacks substrate specificity for glutamine over ammonia and displays a modest activation of the glutaminase domain compared to tbNadE. We report the crystal structures of hsNadE and NAD^+^ synthetase from *M. tuberculosis* (tbNadE) with synthetase intermediate analogues. Based on the observed exclusive arrangements of the domains and of the intra- or inter-subunit tunnels we propose a model for the inter-domain communication mechanism for the regulation of glutamine-dependent activity and NH_3_ transport. The structural and mechanistic comparison herein reported between hsNadE and tbNadE provides also a starting point for future efforts in the development of anti-TB drugs.

## Abstract (web summary)

*M. tuberculosis* NAD^+^ synthetase (tbNadE) is of interest as a drug target. Here the authors present the actively trapped *Homo sapiens* NAD^+^ synthetase (hsNadE) and tbNadE structures and show key differences in the synthetase active site and in structural elements possibly involved in the allosteric regulation of catalysis to be leveraged for the development of *M. tuberculosis* selective inhibitors.

## Introduction

NAD^+^ is the cofactor of enzymes involved in reduction–oxidation reactions but other important roles for it have emerged in recent years in cell signalling, cell division, cell longevity, immune response and cancer^1–6^, and as a possible target in pathogenic bacteria^7,8^ such as *Mycobacterium tuberculosis* (Mtb)^9^. About 1.7 billion people, 23% of the world’s population, are asymptomatically infected with *Mtb* (latent TB) and 5–10% of these will eventually develop active tuberculosis (TB)^10,11^. As such TB remains a global public health problem with very few chemotherapeutic targets validated for extensively drug-resistant TB (XDR-TB) and latent TB^9^. Among these is NAD^+^ synthetase, which catalyzes the last and essential step of *Mtb* NAD^+^ biosynthesis^12^. In humans, NAD^+^ can be synthesized independently of NAD^+^ synthetase^13–15^ making this enzyme attractive for the development of antitubercular drugs.

NAD^+^ synthetase can be either monofunctional ammonia-dependent NAD^+^ synthetase using uniquely free ammonia as a substrate or multifunctional glutamine-dependent NAD^+^ synthetase (^gln^NAD^+^ synthetase) using glutamine as the source of ammonia. The latter belongs to the glutamine amidotransferases (GATs) and has the glutaminase domain of the nitrilase superfamily^16–19^. ^gln^NAD^+^ synthetases are divided in the octameric and homodimeric groups^20^. Herein we focus on the ^gln^NAD^+^ synthetase and on the octameric ones.

**Fig. 1:**
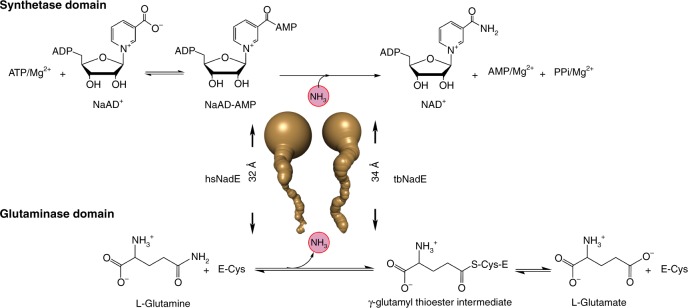
Reaction catalyzed by glutamine-dependent NAD^+^ synthetase. Ammonia tunnels of hsNadE and tbNadE shown in gold indicate the ammonia transfer from the glutaminase to the synthetase active site.

^gln^NAD^+^ synthetase catalyzes the ATP-dependent formation of NAD^+^ from nicotinic acid adenine dinucleotide (NaAD^+^) at the synthetase domain using the ammonia generated from the glutaminase domain^21^ (Fig. 1). The active site coupling of the glutaminase and synthetase domains in prokaryotic and eukaryotic ^gln^NAD^+^ synthetases varies significantly in the degree of glutaminase activation and channeling efficiency. For example, NAD^+^ synthetase in *Mtb* (tbNadE) has the highest degree of glutaminase activation with the maximal channeling efficiency^22^, while inefficient allosteric regulation was reported in *Thermotoga maritima* NAD^+^ synthetase (tmNadE)^23^. In eukaryotes, a compromised glutamine-dependent NAD^+^ synthetase activity was reported in *Saccharomyces cerevisiae* (scNadE) in which roughly 40% of the glutamine is hydrolyzed unproductively, i.e. not forming NAD^+^^24–26^. In scNadE the binding of NaAD^+^ substrate alone at the synthetase active site is sufficient to activate glutaminase activity^25,26^. On the other hand, the glutaminase activation in tbNadE occurs only after the formation of the synthetase intermediate complex (NaAD–AMP and MgPPi)^22^.

Recently, we reported the structures of several complexes of tbNadE showing that coupling between the two active sites is mediated by two active site loops, the synthetase loop named P2 and the glutaminase loop named YRE^22,23,27^. However, in all these complexes the synthetase active site was trapped in an open conformation with the P2 loop disordered and the glutaminase active site was trapped in an inactive conformation with a sub-optimal catalytic alignment of the glutaminase active site residues and with no glutamine bound^22,27^. The molecular mechanism of the allosteric regulation in ^gln^NAD^+^ synthetase has remained elusive in the absence of a crystal structure with the synthetase and glutaminase active sites in an active conformation. Moreover, the absence of structural and kinetic data of hsNadE makes it difficult to leverage any relevant differences with tbNadE for the selective inhibition of the latter. Here, we report the structural and kinetic characterizations of hsNadE and a direct structural comparison of the closed and active conformation of (1) hsNadE structure in complex with a synthetase intermediate analog at 2.84 Å resolution and (2) tbNadE structure in complex with glutamine, sulfonamide derivative 1 (5′-*O*-(*N*-(nicotinyl)sulfamoyl) adenosine; SFI), and PPi at 3.14 Å resolution. Both structures are snapshots of the enzymes in the allosteric state with the synthetase active site loop ordered revealing an exclusive rearrangement of the glutaminase active site and the molecular tunnels. The kinetic and structural characterizations of hsNadE also enable a direct comparison between these two homologs to develop and rationally design inhibitors that selectively inhibit tbNadE.

## Results

### Expression and purification of hsNadE

Due to the failure of hsNadE protein expression in the *E. coli* system used for tbNadE (Supplementary Fig. 1a), the *nadEHs* gene was cloned and overexpressed in Sf9 insect cells with N-terminal SUMOstar and His_6_ tag yielding approximately three times more protein than with N-terminal His_6_ alone (Supplementary Fig. 1b, c). The SUMOstar tag was used for a similar purpose as the regular SUMO tag reported with tbNadE to maximize protein solubility. The recombinant hsNadE protein was purified with a similar purification protocol used for tbNadE^22,27^ but with a higher imidazole concentration (at 50 mM imidazole) to eliminate most non-specific binding proteins in the first Ni-affinity chromatographic step. More than 80% of fusion tag could be cleaved off by SUMOstar protease at a ratio of 1:30 (protease:recombinant protein) at 4 °C. Homogenous protein was obtained after passing through a second Ni-affinity column and gel filtration chromatography with >95% purity on SDS–PAGE (Supplementary Fig. 1d). The final yield was 13 mg/L cell culture.

### Steady-state kinetics and glutaminase activation parameters

A comparison of the steady-state kinetic parameters of hsNadE to our previously reported kinetic data of tbNadE shows that the two homologs are mostly similar but with noteworthy differences in the degree of activation of the synthetase domain and in the specificity of the amino donor substrate. The glutamine substrate specificity between the hsNadE and tbNadE is similar based on *k*_cat_/*K*_m_ (0.45 vs. 0.42 s^−1^ mM^−1^ for NAD^+^ assay and 0.48 vs. 0.4 s^−1^ mM^−1^ for Glu assay for hsNadE (Table 1 and Source Data File) and tbNadE^22^, respectively. The turnover numbers for the ammonia and glutamine-dependent reactions are both six- fold faster for hsNadE (Table 1) than tbNadE^22^. Unlike tbNadE^22^, hsNadE has no specificity to glutamine over ammonia based on *k*_cat_/*K*_m_ (0.45 and 0.49 s^−1^ mM^−1^ for glutamine and ammonia, respectively). The maximal channel efficiency reached under *K*_m_ concentration of glutamine is slightly compromised for the hsNadE compared to the tbNadE (95% in tbNadE^22^ and 85% in hsNadE, Supplementary Table 1 and Source Data File).

**Table 1:** Steady-state kinetic parameters of the hsNadE catalyzed reactions.

Assay	Variable substrate	Fixed substrates	*K*_m_ (mM)	*k*_cat_ (s^−1^)	*k*_cat_/*K*_m_ (s^−1^ mM^−1^)
Glutamine-dependent reaction					
NAD^+^	NaAD^+^	Gln, ATP	0.26 ± 0.04	3.2 ± 0.1	12 ± 2
NAD^+^	ATP	Gln, NaAD^+^	0.17 ± 0.02	3.79 ± 0.07	22 ± 2
NAD^+^	Gln	NaAD^+^, ATP	8.3 ± 0.6	3.72 ± 0.07	0.45 ± 0.03
Glu	Gln	NaAD^+^, ATP	11.6 ± 0.7	5.6 ± 0.1	0.48 ± 0.03
Glu	Gln	None	34 ± 6	0.18 ± 0.01	0.005 ± 0.001
Ammonia-dependent reaction					
NAD^+^	NaAD^+^	NH_3_, ATP	0.52 ± 0.04	23.4 ± 0.5	45 ± 4
NAD^+^	ATP	NH_3_, NaAD^+^	0.22 ± 0.03	18.5 ± 0.5	84 ± 14
NAD^+^	NH_3_	NaAD^+^, ATP	42 ± 7	20 ± 1	0.49 ± 0.08

The glutaminase activity in hsNadE is enhanced by the formation of the NaAD–AMP intermediate and MgPPi or by the binding of the NaAD–AMP intermediate analog (NaAD^+^ and AMP) and MgPPi as in tbNadE but the magnitude of this activation is significantly less (Table 2 and Source Data File, 179-fold vs. 31-fold and 44-fold vs. 12-fold for synthetase intermediate complex and intermediate analog, respectively). The glutaminase activity of tbNadE in the presence of SFI, an analog of NaAD–AMP, was measured in combination with different ligands in order to test the ability of SFI to trap the glutaminase domain in an active conformation. The addition of pyrophosphate induces a 16-fold activation of the glutaminase domain by 16-fold (Table 2). This data suggests that the occupancy of both SFI inhibitor and MgPPi could mimic the formation of synthetase intermediate complex (NaAD–AMP and MgPPi) and activate glutaminase activity at moderate level.

**Table 2:** Glutaminase activation of hsNadE and tbNadE.

Enzyme	Effectors	Sample reactions	*k*_cat_ (s^−1^)	Fold activation
hsNadE	None	Glutamine	0.180 ± 0.01	--
	NaAD^+^, ATP/Mg^2+^	Glutamine	5.6 ± 0.1	31
	NaAD^+^	Glutamine	0.316 ± 0.001	1.8
	NAD^+^	Glutamine	0.214 ± 0.002	1.2
	ATP/Mg^2+^	Glutamine	0.091 ± 0.002	No activation
	PPi/Mg^2+^	Glutamine	0.185 ± 0.002	No activation
	NAD^+^, ATP/Mg^2+^	Glutamine	0.113 ± 0.006	No activation
	NaAD^+^, AMP/Mg^2+^	Glutamine	0.375 ± 0.001	2.1
	NaAD^+^, AMP,PPi/Mg^2+^	Glutamine	2.2 ± 0.4	12
tbNadE	None	Glutamine	0.0038 ± 0.0002	–
	NaAD^+^, ATP/Mg^2+^	Glutamine	0.68 ± 0.03	179^a^
	NaAD^+^, AMP,PPi/Mg^2+^	Glutamine	0.167 ± 0.007	44^b^
	SFI	Glutamine	0.0021 ± 0.0002	No activation
	SFI, PPi/Mg^2+^	Glutamine	0.061 ± 0.004	16

^a^Kinetic data reported in LaRonde-LeBlane et al. (2009)^22^

^b^Kinetic data reported in Chuenchor et al. (2012)^27^

### Overall structure of hsNadE

**Fig. 2:**
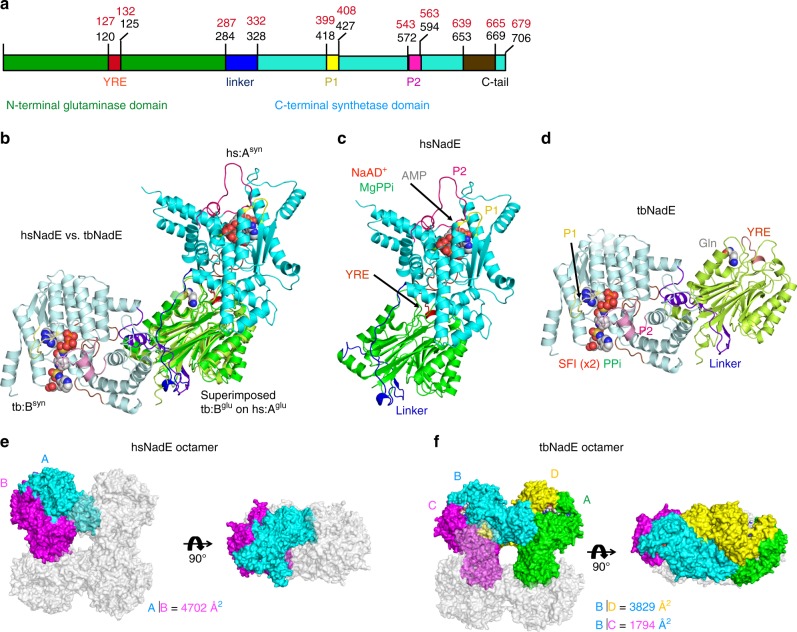
Structures of hsNadE and tbNadE in the activated complexes. **a**–**d** Diagram and structures of the glutaminase and synthetase domains in one polypeptide chain of the superimposed structure (**b**) of hsNadE with NaAD^+^, AMP, and MgPPi (**c**) and tbNadE with the sulfonamide derivative 1 (SFI) and PPi (**d**) using the glutaminase domains alone for superimposition. Residue numbering in the schematic diagram of hsNadE and tbNadE is shown in black and red, respectively. The P1 and P2 loops at the synthetase active sites, the YRE at the glutaminase domain, and the linker connecting the glutaminase and synthetase domains are colored accordingly to the diagram. Ligands bound in the structures are shown in spheres. **e**, **f** Comparison of the molecules in the asymmetric unit and in the subunit interfaces in hsNadE (**e**) and tbNadE (**f**). The buried surfaces of both complexes are compared between subunits A and B in hsNadE, subunits B and D, and subunits B and C in tbNadE. The number was calculated by PISA^28^ and shown in Å.

**Fig. 3:**
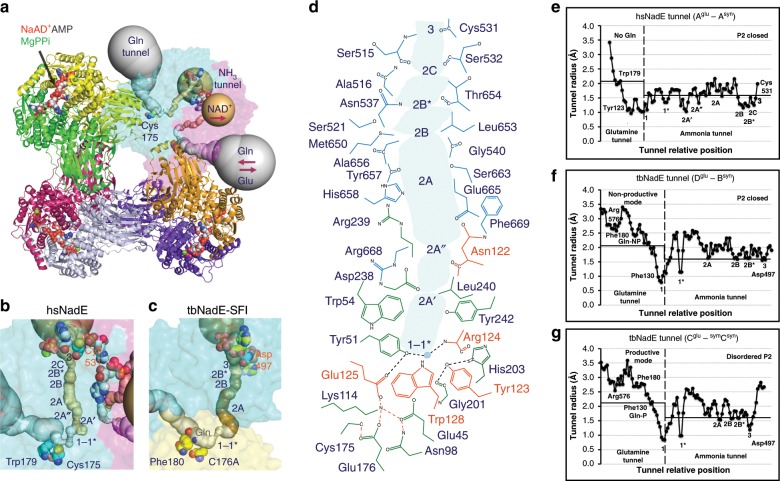
Octameric assembly and molecular tunnels connecting the two active sites in hsNadE. **a** The biological unit of hsNadE is generated by crystal symmetry as an octamer. The glutamine and ammonia tunnels in subunits A and B are in gray and golden, respectively. The red arrow indicates the direction of the substrate/product flows in/out the molecular tunnel. **b**, **c** Comparison of the constrictions located in the molecular tunnels found in hsNadE (**b**) and tbNadE-SFI complexes (**c**) within the single subunit A and two subunits D^glu^–B^syn^, respectively. Key residues (glutamine-binding residue, catalytic cysteine, and last position of the ammonia transport) are shown as spheres. **d** Polar, nonpolar, and charged residues forming the ammonia tunnel and constrictions in the hsNadE complex. The residues lining the ammonia tunnel belong to the glutaminase domain (green), the YRE loop (orange), and the synthetase domain (cyan). A water molecule coordinated with the residues forming constriction 1-1* and the YRE loop residues is shown in gray sphere with a light blue contour and its position indicates the start of the ammonia transport. **e**–**g** Ammonia tunnel radii calculated in subunit A of hsNadE complex (**e**), in subunits D^glu^–B^syn^ (**f**), and in subunits C^glu^-^sym^C^syn^ (**g**) of the tbNadE–SFI complex. The dashed line at constriction 1-1* shows the boundary of the glutamine and ammonia tunnels. 2.1 and 1.6 Å cut-off radii are used for the glutamine and ammonia tunnels, respectively. Location of the glutamine-binding site is defined by Tyr123^HS^/Phe130^TB^ and Trp179^HS^/Phe180^TB^. The last position of ammonia in the ammonia tunnel is marked by Cys531^HS^/Asp497^TB^.

We co-crystalized wild type hsNadE with NaAD^+^ and AMP (the synthetase intermediate analog), and MgPPi, which activates the glutaminase activity in hsNadE by 12-fold (Table 2), to obtain the crystal structure of the hsNadE in the closed conformation of the synthetase active site and with the activated glutaminase active site. The co-crystals of hsNadE crystallized in needle-like shape diffracted up to 2.84 Å resolution (Supplementary Table 2 and Supplementary Fig. 1e, f). The core structure of the glutaminase and synthetase domains of hsNadE is similar to that of tbNadE, despite sharing only 23% amino acid sequence identity (Fig. 2a–d and Supplementary Fig. 2a, b). Structural superimposition between hsNadE and tbNadE for each domain shows more differences between the synthetase domains than the glutaminase domains (Supplementary Fig. 3a, b; r.m.s.d. of 1.1 for 205 Cα carbon for the glutaminase domain, whereas r.m.s.d. of 3.5 for 156 Cα carbon for the synthetase domain). By superimposing the glutaminase domain of both homologs, the synthetase domain of hsNadE rotates by 103° with respect to the synthetase domain of tbNadE (Fig. 2b, Supplementary Fig. 3c). The linker in hsNadE (blue ribbon) positions the synthetase active site closer to the glutaminase active site within the same subunit than in tbNadE (43 Å in hsNadE and 59 Å in tbNadE, Supplementary Fig. 4a, b). Some structural elements of the synthetase domain, such as α10′, α10″, α21, C-tail loop, were truncated out for a structural superimposition of ligands bound in the synthetase active site (r.m.s.d. of 1.1 for 100 Cα carbon, Supplementary Fig. 3d, e). Two molecules are present in the asymmetric unit as a homodimer, whereas a tetramer was found in tbNadE structure (Fig. 2e, f). The buried surface between the homodimer is 4702 Å^2^ (calculated by PISA^28^), whereas the buried surface between the two corresponding subunits in tbNadE is only 1794 Å^2^. Similarly to tbNadE, the biological unit of hsNadE is an octamer as shown by gel filtration chromatography. The biological unit is generated by symmetry-related molecules from the crystal structure (Figs. 2e, f, 3a, Supplementary Fig. 4c). Double layers of tetrameric glutaminase are formed as a core with eight decorating synthetase units at the four edges (Fig. 3a).

### Intra-subunit molecular tunnel in hsNadE

Analysis with the Caver program reveals an intra-subunit tunnel forming within a chain in hsNadE structure (Fig. 3a, b, d, e and Supplementary Fig. 4a) unlike the inter-subunit tunnel found in tbNadE (Fig. 3c and Supplementary Fig. 4b). The glutamine and ammonia tunnels are 20 and 32 Å in length, respectively. Both tunnels have mixed hydrophobic and hydrophilic characters (Fig. 3d). The tunnels are composed of either the backbone or the side chain of polar, nonpolar, and charged-amino acid residues similarly to those in tbNadE^22,27^. However, the average tunnel radius of the hsNadE is much more constricted (~1.5 Å, Fig. 3e) than that of the tbNadE complex herein reported (Fig. 3f, g) and those previously reported (~2 Å)^27^. The ammonia tunnel is also sealed off at both ends and the glutamine-binding pocket is inaccessible in hsNadE. Radius widening in the ammonia tunnel during the catalytic cycle was expected but the dynamics of the system could have been limited in the crystal structure due to the cryocooling^29^ and to the short-lived nature of particular conformations.

Of the constrictions previously observed in the ammonia tunnels of several tbNadE complexes^22,27^, constrictions 1, 1*, 2A, 2B, 2B*, 2C, and 3 are conserved in hsNadE (Fig. 3b, c). The biggest difference between these share constrictions is seen in constriction 1, a major bottleneck of the glutamine tunnel. This constriction in hsNadE is formed by a hydrophilic patch of glutaminase active site residues (Cys175^HS^, Glu45^HS^, Glu176^HS^) and nearby residues (Gly201^HS^, Ser202^HS^) and by a hydrophobic patch formed by Trp54^HS^, Tyr51^HS^, Tyr242^HS^, Leu240^HS^, Tyr123^HS^ (Fig. 3d). Constriction 1 in hsNadE, contrary to what it has been observed in all tbNadE structures, extends from the border between the two tunnels much more towards the glutaminase active site (Fig. 3e–g). Another major difference in the hsNadE tunnels is the presence of two additional constrictions, 2A′ and 2A″ located between constrictions 1-1* and 2A (Fig. 3b–g). Constriction 2A′ is formed by a mix of hydrophobic and hydrophilic residues of Trp54^HS^, Leu240^HS^, Asn122^HS^ (backbone), and Asp238^HS^ (backbone and side chain). Constriction 2A″ also contains a hydrophobic/hydrophilic patch formed by Arg668^HS^ (backbone), Arg239^HS^ (backbone), Leu240^HS^, and Asn122^HS^ (side chain). The remainders of the constrictions (2A, 2B, 2B*, 2C) are similarly formed by a mix of hydrophobic and hydrophilic residues listed in Fig. 3d. Unexpectedly, the last site for the ammonia transfer reported in tbNadE (Asp497^TB^) is largely offset compared to corresponding residue (Asp530^HS^) in hsNadE. Instead of and adjacent to the strictly conserved Asp530^HS^, Cys531^HS^ forms constriction 3, the last site for the ammonia transfer in hsNadE.

### NaAD^+^ and ATP-binding sites at the hsNadE synthetase domain

**Fig. 4:**
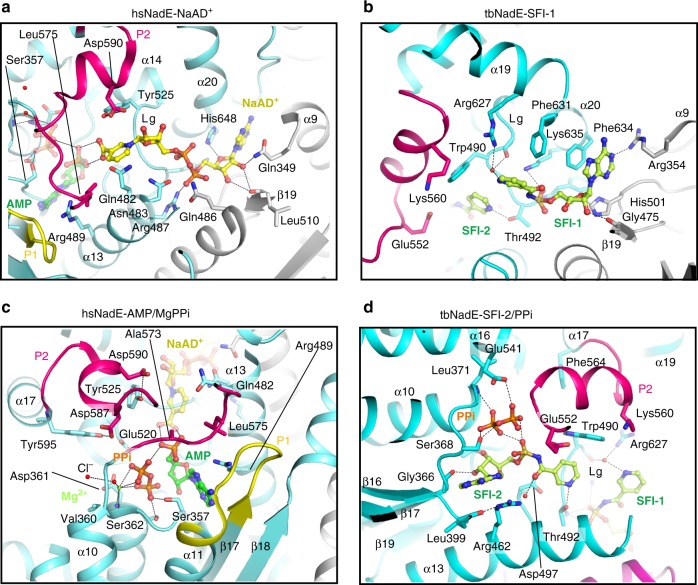
Structural basis of substrate specificity of the synthetase domain. **a**, **b** Binding of the NaAD^+^ (yellow stick, **a**) and sulfonamide derivative 1 (light green stick, SFI, **b**) in NaAD^+^-binding site of hsNadE and tbNadE, respectively. **c**, **d** Binding of the AMP, MgPPi (green/orange stick, green sphere) in hsNadE (**c**) and SFI, PPi in tbNadE (**d**). Cl^−^ atom hydrogen bonds to Mg^2+^ is in red sphere. In the tbNadE–SFI complex, the first SFI molecule is bound in the NaAD^+^-binding site (**b**) and the second SFI together with PPi is bound in the ATP-binding site (**d**) in a similar manner than the synthetase intermediate analog. This results in the ordering of the P2 loop (pink residues).

Like other ammonia-dependent and glutamine-dependent NAD^+^ synthetases, the NaAD^+^-binding site of hsNadE is formed at the dimer interface and is exposed to solvent (Fig. 4a and Supplementary Fig. 5a)^16,22,30^. The imidazole ring of His648^HS^ hydrogen bonds to the adenine ribosyl oxygen of NaAD^+^ and makes hydrophobic contact to the adenine base, while the corresponding residue in tbNadE, Phe634^TB^ (Fig. 4b), stabilizes the adenine base by hydrophobic interaction only. The residue stabilizing the nicotinic moiety is Tyr525^HS^ (a well-conserved residue among ^gln^NAD^+^ synthetase, except for tbNadE, where it is replaced by Trp490^TB^, Fig. 4b, Supplementary Fig. 2b). The hydrophobicity of tyrosine and tryptophan side chains is chemically conserved to stabilize nicotinic ring via π-interaction. However, the smaller side chain and the presence of hydroxyl group of the conserved tyrosine in other ^gln^NAD^+^ synthetases, but not in tbNadE, may contribute to the higher specificity constant with NaAD^+^ compared to tbNadE (Table 1)^22,23^.

In the ATP-binding site of hsNadE (Fig. 4c and Supplementary Figs. 5b and 6a, b), AMP and MgPPi are bound in a deep cleft as reported in the tbNadE structure (Supplementary Fig. 5c)^27^ with one Mg ion coordinated to three O atoms of PPi, to the main chains of ATP-binding site residues including Val360^HS^ and Asp361^HS^ (ATP pyrophosphate fingerprint), and a Cl^−^ atom (red sphere, Fig. 4c). In this hsNadE complex, the ATP-binding site is fully protected by the closure of the P2 loop (Supplementary Figs. 5a, 7a–d) via three crucial contacts with the ligands and with the synthetase residues. First, Tyr525^HS^ makes hydrophobic contact with the nicotinic ring and hydrogen bonds to Asp590^HS^, a P2 loop residue (Fig. 4a, c). These two residues are replaced by Trp490^TB^ and Lys560^TB^, respectively, in tbNadE (Fig. 4b, d). The substitution of Tyr525^HS^ with Trp490^TB^ results more likely in an increased flexibility of P2 loop in tbNadE compared to hsNadE due to the absence of the hydrogen bond between the hydroxyl group of Tyr525^HS^ and P2 loop residue. Second, Leu575^HS^ and Ala573^HS^, P2 loop residues, make hydrophobic contacts with the nicotinic moiety of NaAD^+^ and the α-phosphate of AMP (Fig. 4a, c). Third, Tyr595^HS^, a residue on the N-terminal α17, hydrogen bonds with Asp587^HS^, a P2 loop residue.

### P2 loop closure in tbNadE

As predicted from the kinetic data showing 16-fold activation of the glutaminase domain when the synthetase domain is occupied by SFI and MgPPi, co-crystallization of tbNadE with SFI, MgPPi, and glutamine trapped the enzyme in the closed activated conformation. In this crystal structure, we found one SFI molecule occupying the NaAD^+^-binding site and another SFI along with PPi bound in ATP-binding site (SFI-1 and SFI-2, respectively, Fig. 4b, d). The SFIs exploit the same hydrophobic and hydrophilic contacts as NaAD^+^-AMP in the previously reported tbNadE complex with NaAD^+^, AMP, PPi structure (PDB code 3SZG, Supplementary Fig. 5d, e)^27^. Closure of the P2 loop observed in subunit B^syn^ reveals two close contacts, the first between the P2 loop residue Glu552^TB^ and *N*-(nicotinyl)sulfamoyl linkage of SFI bound in the ATP-binding site (Fig. 4d, SFI-2), and the second between the one O atoms of PPi and Glu541^TB^, a terminal residue of α16 adjacent to P2 loop (Fig. 4d and Supplementary Fig. 5e). The nicotinic ring like moiety of the SFI bound in the NaAD^+^-binding site is highly flexible due to the absence of protein–ligand interactions (Fig. 4b, Supplementary Fig. 6c, d). PPi was built only in the synthetase active site of subunit B with the P loops ordered (partial occupancy of 0.58, Supplementary Figs. 8a–d, 7e–h). The binding of SFI in the ATP-binding site and MgPPi at the synthetase active site leads to the ordering of the P2 loop (in subunit B^syn^, Fig. 4d, Supplementary Fig. 5e, f) and rearrangement of the glutaminase active site to trap in a non-productive mode (Gln-NP) the glutamine substrate in its coupled active site (in subunit D^glu^, Fig. 3f, Supplementary Fig. 5g). Interestingly, the ordering of the P loops in subunit B^syn^ (and ^sym^B^syn^ in octamer) could induce a productive binding mode of glutamine (Gln-P) activating the glutaminase active site in the uncoupled active site (C^glu^ and ^sym^C^glu^ Fig. 3g, Supplementary Fig. 5g). No Mg^2+^ could be identified in this complex due to a partial occupancy of PPi. The binding of SFIs and PPi reduces by a magnitude the atomic fluctuation calculated by DynaMut^31^ at the P1 and P2 loops that become ordered in subunit B^syn^ compared to the atomic fluctuation in the P loops of hsNadE complex (Supplementary Fig. 9a, b).

### Activation of and tunnel gating in the glutaminase domain

**Fig. 5:**
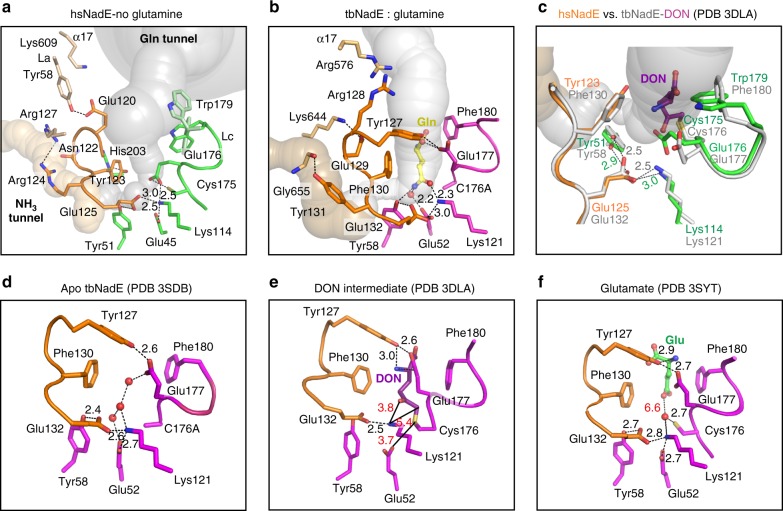
Role of the YRE loop and arrangement of the glutaminase active site. **a** This panel illustrates the hsNadE glutaminase domain with the glutaminase active site free of ligand in a complex with the synthetase active site fully occupied with the intermediate analog. **b** The glutaminase active site of tbNadE subunit D is occupied with glutamine in a complex with SFIs and PPi bound in the synthetase active site of the coupled subunit B. The YRE loop residues are shown in orange sticks in all complexes, the catalytic and substrate-binding residues are shown in green in hsNadE and purple in tbNadE. The glutamine substrate and water in the ammonia tunnel of the tbNadE complex are shown as a yellow stick and red sphere. **c** Overlay of the hsNadE and tbNadE–DON complexes (PDB code 3DLA). Distance of hydrogen bonding between (i) YRE loop’s glutamate and catalytic lysine and (ii) the glutamate and constriction 1’s tyrosine is shown as green (hsNadE) and gray (tbNadE) numbers. **d**–**f** The glutaminase active site of previously reported tbNadE structures bound to waters in the apo form (PDB code 3SDB) (**d**), with DON bound (dark purple stick, PDB code 3DLA) (**e**), and with glutamate bound (green stick, PDB code 3SYT (**f**).

The catalytic residues at the glutaminase domain involved in glutamine hydrolysis of the enzymes of the nitrilase superfamily are the nucleophilic cysteine (Cys175^HS^, Cys176^TB^), the acid/base glutamate (Glu45^HS^, Glu52^TB^), and the catalytic lysine (Lys114^HS^, Lys121^TB^)^22,32–34^. Based on previously reported mutational studies^35,36^, a second glutamate adjacent to the C–E–K triad was found to be essential for enzymatic activity and to play a key role in positioning productively the substrate^36,37^ in some members of nitrilase superfamily. A structural comparison of the C–E–K triad of a handful of available crystal structures from six subfamilies of the nitrilase superfamily (Supplementary Fig. 10a–i) shows that this second glutamate (Glu125^HS^ and Glu132^TB^ in the structures reported herein) contributes to the stabilization of the loop near the catalytic triad (called YRE loop in ^gln^NAD^+^ synthetase) and of the optimal side chain geometry of the catalytic lysine^36–41^. Two hydrogen bonds are involved between the glutamate O^ε2^ and tyrosine O^η^ or histidine N^δ1^, and the glutamate O^ε1^ and catalytic lysine N^ζ^ for the former and latter roles. In our reported crystal structures, the hydrogen bonds are fine-tuned during the catalytic cycle between (i) this second glutamate (Glu125^HS^ and Glu132^TB^) and a conserved Tyr in eukaryotic NAD^+^ synthetases (Tyr51^HS^ and Tyr58 ^TB^) and (ii) this second glutamate (Glu125^HS^ and Glu132^TB^) and catalytic lysine (Lys114^HS^, Lys121^TB^) (Fig. 5a–f). Without this glutamate, the YRE loop and the side chain of lysine are more flexible (high-temperature factors) or disconnected as also shown by mutagenesis and structures of the amidase enzyme of the nitrilase superfamily^36^ (Supplementary Fig. 10e). This second glutamate as discussed in the next sub-section also contributes to constriction 1, the major bottleneck of the ammonia tunnel.

In the absence of a ligand at the glutaminase active site of hsNadE complex, the Glu176^HS^ side chain moves 78° towards the Cys175^HS^–Glu45^HS^–Lys114^HS^ triad compared to the herein described tbNadE complex and the other previously reported complexes (Fig. 5a–f, Supplementary Fig. 11a)^22,27^. The α-carboxyl groups of Glu176^HS^ shifts by 4.3–4.6 Å toward the catalytic triad to enhance the nucleophilicity of the cysteine thiol and to stabilize the catalytic lysine residue. However, this inward movement of Glu176^HS^ side chain collapses the oxyanion hole, which stabilizes the negative charge of the tetrahedral γ-glutamylthioester intermediate reported in tbNadE–DON complex (Fig. 5c, e)^22^. In addition, this conformation of the Glu176^HS^ side chain blocks the glutamine tunnel and clashes with the glutamine molecule as shown in the overlay with the tbNadE–glutamine–SFI–PPi structure (Supplementary Fig. 11a). This suggests the presence of a gating system at the glutamine tunnel by swinging of the glutamate side chain located at the catalytic triad.

A second gating system in the hsNadE structure is found at the entry way of the glutamine tunnel. The side chain of Trp179^HS^ (corresponding to Phe180^TB^ in tbNadE) adopts multiple conformations in two subunits (Fig. 5a, one conformation in subunit A; dark green, and other two conformations in subunit B; light green). The conformation found in subunit A yields to the most restricted entry of the glutamine tunnel (1.97 Å) likely blocking the incoming glutamine (Fig. 3e), while a conformation found in subunit B widens the tunnel (3.03 Å, Supplementary Fig. 6a). Interestingly, in hsNadE Trp179^HS^-subunit A is observed in a conformation that restricts the entrance to the glutamine tunnel, and Glu176^HS^ is positioned to prevent glutamine binding (Fig. 5a). Flexibility of the Trp179^HS^ side chain in the absence of glutamine binding suggests an additional gating system with Glu176^HS^ regulating the entry of glutamine to the glutaminase active site. This gating mechanism by the corresponding residues in tbNadE (Phe180^TB^ and Glu177^TB^) has not been observed in any of the tbNadE structures, including the structure reported herein where the access to the glutamine tunnel is always open (Fig. 5b–f).

### YRE loop at the glutaminase domain

**Fig. 6:**
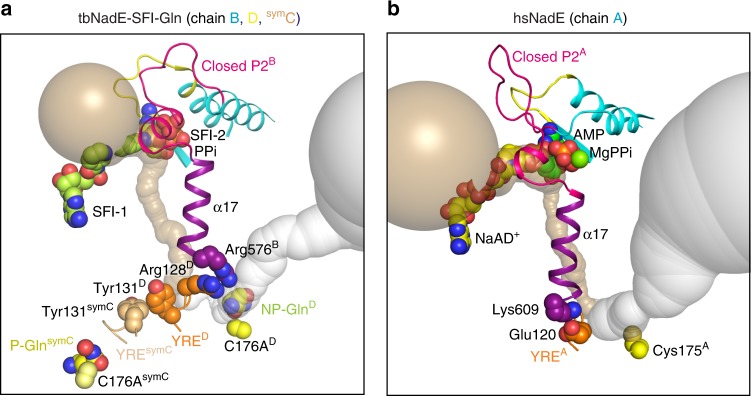
Comparison of the regulatory elements between the tbNadE and hsNadE complexes. **a**, **b** Cartoon structures of the closed P2 loop (cyan), the α-17 helix (purple), and the YRE loop (orange for the coupled subunit, and brown for the uncoupled subunit) in tbNadE–SFI complex bound to glutamine (**a**) and hsNadE (**b**). Panel **a** shows the α-17 helix connecting the closed P2 loop of B^syn^ to the YRE loop of the coupled subunit D^glu^. This YRE loop makes also contact with the YRE loop of the uncoupled subunit ^sym^C thus resulting in a productive binding of the glutamine substrate in ^sym^C of tbNadE. Panel **b** shows the α17 connects the closed P2 and YRE loops with the same subunit (chain A) in hsNadE. The pairs of Arg128^TB^/Arg576^TB^ and Glu120^HS^/Lys609^HS^ connecting the YRE and α-17 helix of the coupled subunits in tbNadE and hsNadE, respectively, are shown as orange and purple spheres. The Tyr131^TB^/^symC^Tyr131^TB^ in tbNadE are shown as orange and brown spheres, respectively. The catalytic cysteine and the C176A variant in the glutaminase active site are shown as yellow spheres. The Caver calculated ammonia and glutamine tunnels are shown in golden and gray, respectively.

The crucial YRE loop regulatory element was previously reported based on crystal structures^22^, phylogenetic tree, and multiple amino acid alignment of other prokaryote and eukaryote NAD^+^ synthetases^23^. Six residues of YRE loop in hsNadE starting from Glu120^HS^ to Glu125^HS^ (Tyr127^TB^ to Glu132^TB^ in tbNadE) and five of the six amino acid residues of this loop are different between hsNadE and tbNadE (Supplementary Fig. 2a). However, the YRE loop retains among the homologs four major roles of regulation during the catalytic cycle: (1) interaction with either the incoming glutamine substrate, ES covalent intermediate, or outgoing glutamate product; (2) regulation of the major bottleneck of constriction 1-1* at the glutamine and ammonia tunnel border during glutamine hydrolysis; (3) regulation of the ammonia transport in the ammonia tunnel through the series of constrictions 2; and (4) communication with the synthetase active site. First, the YRE loop residue interacts with glutaminase active site ligand. In three different complexes of tbNadE bound to DON, glutamate, and glutamine, Tyr127^TB^ interacts with the glutaminase active site ligand and the carboxyl oxygen of Glu177^TB^ (Fig. 5b, e, f). In the hsNadE complex with no glutaminase active site ligand, Glu120^HS^ moves outward to hydrogen bond with Tyr58^HS^ of the glutaminase active site of the adjacent subunit (Fig. 5a). This tyrosine is exclusive to hsNadE and is replaced by leucine in the other glutamine-dependent NAD^+^ synthetases (Supplementary Fig. 2a). Second, the YRE loop regulates an entry way of the ammonia tunnel by forming the major bottleneck at constriction 1 with other constriction residues. These YRE residues are Tyr123^HS^/Glu125^HS^ in hsNadE and Phe130^TB^/Glu132^TB^ in tbNadE. Other residues forming constrictions 1 are His203^HS^/Tyr51^HS^/Gly201^HS^ in hsNadE and Pro204^TB^/Tyr58^TB^ in tbNadE. Interestingly, Tyr123^HS^ and His203^HS^ are conserved in eukaryotes, whereas Phe130^TB^ and Pro204^TB^ are conserved in prokaryotes (Supplementary Fig. 2a). This suggests that these residues might be responsible for the different enzyme mechanisms in eukaryotic and prokaryotic ^gln^NAD^+^ synthetase. For example, constriction 1 in hsNadE is stabilized by hydrogen bonds and hydrophobic contacts, while the corresponding region in tbNadE is stabilized only by hydrophobic interactions. Third, the YRE loop itself forms or interacts with residues of constrictions 2 (2A, 2A′, and 2A″ in Fig. 3b–d). The polar side chain and backbone of Asn122^HS^ in hsNadE form constrictions 2A′ and 2A″, respectively, and the charged side chain of Glu129^TB^ hydrogen bonds to Lys644^TB^, the residue forming constriction 2A in tbNadE^27^. Fourth, YRE loop links to the remote synthetase active site via α17 and P2 loop. One end of this helix connects to the YRE loop at the glutaminase active site and the other end connects to the P2 loop at the synthetase active site of the subunit that is paired by the same ammonia tunnel. The inter-subunit tunnels in tbNadE seems to use the pair of Arg128^TB^ and Arg576^TB^ from the YRE and α17, respectively, for communicating between the two coupled active sites (Fig. 6a). Both arginines are unique for tbNadE among the ^gln^NAD^+^ synthetase (Supplementary Fig. 2a, b). The intra-subunit tunnels in hsNadE mediate the connection between YRE loop and α17 through Lys609^HS^ and Glu120^HS^ (Fig. 6b). This latter residue is either conserved or chemically conserved in eukaryotic ^gln^NAD^+^ synthetase (Supplementary Fig. 2).

## Discussion

The few kinetic and structural studies of ^gln^NAD^+^ synthetases have shown that these enzymes are complex multifunctional enzymes displaying catalytic coupling between two reactions^22,23,26,27^. Only two enzymes of this class have been previously structurally characterized, the octameric tbNadE^22,27^ and the homodimeric enzyme from *Burkholderia thailandensis*^20,42^, revealing a molecular tunnel connecting the two active sites and the identification of some structural elements possibly involved in the active site coupling. However, the molecular mechanism of the allosteric regulation remained elusive, since these structures represented the enzymes in an inactive conformation^20,22,27^. We here propose a model for the inter-domain communication mechanism enabled by the activated structures of tbNadE and hsNadE herein described. Additionally, considering that NAD^+^ synthetase has been validated as an anti-TB drug target^12^, these structures provide long-sought data for drug development against active and latent TB.

Based on the structural analysis herein reported and the mutagenesis data previously reported for tbNadE^22^, we propose different coupling mechanisms between the synthetase and glutaminase activities within the oligomeric unit in tbNadE and hsNadE. First, the ammonia tunnel in tbNadE links the glutaminase and synthetase active sites between two subunits while in hsNadE it connects active sites within the same subunit. Second, the activation of the glutaminase domain and ordering of the P2 loop at the synthetase active site of tbNadE do not occur simultaneously between two coupled subunits and the ordering of the P2 loop of one subunit. Ordering of P2 loop leads to the activation of the glutaminase active site of an uncoupled-tunnel subunit in the same octamer. This differs in hsNadE where the ordering of P2 loop after the NaAD–AMP intermediate formation occurs simultaneously in all subunits with the same state of glutaminase active sites in the octamer. Third, different residues on YRE loop, P2 loop, and connecting modules are responsible for active sites’ communication between the two enzymes. These differences may contribute to the different degrees in glutaminase activation and channeling efficiency between tbNadE and hsNadE.

Active site coupling in tbNadE is evident in both enzymes by the observed high activation of the glutaminase domain upon formation of the acyladenylate intermediate (NaAD–AMP) at the synthetase active site^8^. As mentioned earlier, using SFIs and MgPPi we were able to obtain the structure of the activated conformation of tbNadE with an ordered P2 loop at the synthetase active site and the glutaminase active site in a closed and active conformation. In this tbNadE complex the glutamine substrate is trapped in the active site of four out of eight subunits (chain C, D, ^sym^C, and ^sym^D). The productive binding mode of glutamine in subunits C and ^sym^C is evident by the position of the glutamine in the innermost catalytic center, while the unproductive binding mode in subunits D and ^sym^D is similar to the glutamate product binding mode in the previously reported tbNadE structure (PDB code 3SYT, Supplementary Fig. 11b)^27^. For example, the activation of the glutaminase domain of subunit ^sym^C is not triggered by the ordering of the P2 loop in the synthetase active site of the tunnel-linked subunit C but by the ordering of the P2 loop in the synthetase active site of B. As Fig. 6a shows, ordering of P2 loop of B^syn^ activates ^sym^C^glu^ likely via (i) the N-terminus Arg576 of α17 interacting with Arg128 of the YRE loop of D^glu^, and (ii) the close contact of Tyr131^TB^ at the opposite end of the YRE loop of D^glu^ with the corresponding residue in ^sym^C^glu^. Differently, in the active conformation of hsNadE the synthetase active site links to the glutaminase active site within a single subunit via Tyr595^HS^ residue on the N-terminal α17 end and Lys609^HS^ located on the α17 C-terminus that closed contacts Glu120^HS^ YRE loop residue (Fig. 6b).

**Fig. 7:**
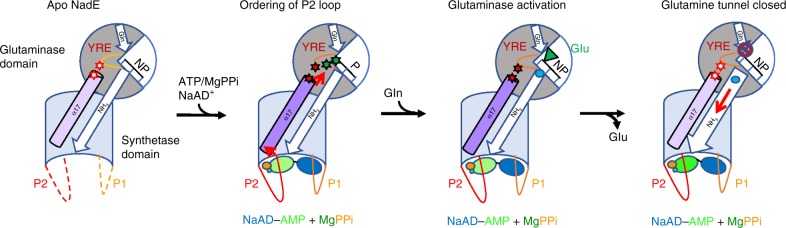
Proposed shared steps in hsNadE and tbNadE for the glutaminase activation. Dash and solid curved lines represents disorder and ordered loops, respectively. NP and P indicate a non-productive and a productive glutaminase active site, respectively. Light and dark purple coloring of α-17 indicate the absence or presence of activating interactions with the YRE loop. Similarly, white and color filled stars represent the absence or presence of interactions between α-17 and YRE loop (in red), and YRE and the catalytic triad (in green). Orange, green, dark blue, and light blue spheres represent PPi, the AMP, and NaAD^+^ moiteties of NaAD–AMP, and ammonia. The green filled triangle represents glutamate. The “no access” sign shows the gating mechanism that blocks access to the glutamine tunnel. The addition of Gln is shown in the model after formation of the NaAD–AMP for simplicity but the order of its addition remains unclear.

Despite these differences in the two active conformations of tbNadE and hsNadE, the structural similarities observed allow us to propose that the activation of the glutaminase domain with the formation of NaAD–AMP intermediate undergoes through these shared steps (Fig. 7). (1) An initial step of closure of the P2 loop is induced by the binding of both NaAD^+^ and MgATP. (2) Upon ordering of the P2 loop, the NAD-adenylate intermediate is formed and induces side-chains rearrangements of regulatory modules, such as α17 and YRE loops coupling the two remote active sites. In the glutaminase active site these structural rearrangements results in the observed interaction between YRE loop’s glutamate and the catalytic lysine thus stabilizing the productive orientation of the C–E–K triad (i.e. activating the glutaminase active site). (3) The productive conformation of the glutaminase active site causes the glutamine hydrolysis to occur, ammonia is released and glutamate moves outward to a non-productive-binding site. (4) The glutamine tunnel is narrowed and closed off by swinging movement of a double module of Trp179^HS^ and Glu176^HS^. Then ammonia is transferred to the synthetase active site within the sealed molecular tunnel. However, it remains still unclear when exactly glutamine binds and when the widening of each constriction along the glutamine tunnel occurs to unblock the ammonia to transfer to the synthetase active site.

**Fig. 8:**
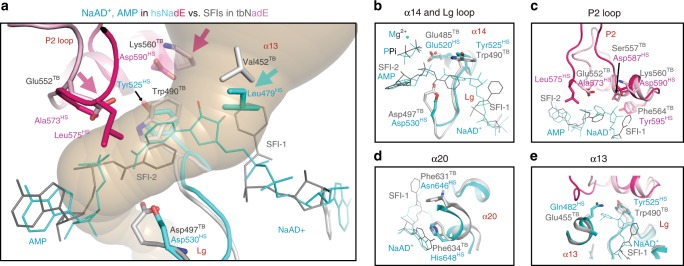
Implication for drug design specific to tbNadE. **a** Overlay of tbNadE and hsNadE complexes. NaAD^+^ shown with AMP in hsNadE in cyan and SFIs in tbNadE in gray (PPis are omitted for clarity). The P2 loop residues are pink and light pink in hsNadE and tbNadE, respectively. Residues important for future anti-TB drug design are shown as sticks. Pink and cyan arrows highlight the residue with the biggest difference in size and/or character between homologs. **b**–**e** Comparison of the synthetase-binding site between homologs at α14 and the Lg loop (**b**), P2 loop (**c**), α20 (**d**), and α13 (**e**).

The analysis herein described is significant for future efforts in the development of an anti-TB drug. Previously, the crystal structure of tbNadE bound to NaAD^+^ (PDB code 3DLA) was used for docking studies of 118 urea–sulfonamide analogs^43^ but this structure has a fully exposed synthetase active site with the P2 loop opened. Based on the structural and sequence differences in the synthetase active site of tbNadE and hsNadE herein shown, a potent tbNadE inhibitor should leverage the differences in the NaAD^+^-binding residues, such as Lg loop (Trp490^TB^), α20 (Phe631^TB^ and Phe634^TB^), and α13 (Glu455^TB^ and Val 452^TB^), (ii) the P2 loop residues (Phe564^TB^, Lys560^TB^, Ser557^TB^, and Glu552^TB^) (Fig. 8a–e). The overlay of the tbNadE and hsNadE structures (pink arrows in Fig. 8a) suggests the design of inhibitors engaging Lys560^TB^ and Glu552^TB^ P2 loop residues to stabilize the NAD-adenylate intermediate analog. In conclusion, the differences herein highlighted in the synthetase active site, the ammonia tunnel, and the allosteric regulation of hsNadE and tbNadE are excellent advantage starting points for future efforts in the design of inhibitors selective for tbNadE.

## Methods

### Cloning and bacmid preparation of hsNadE

The cDNA sequence of hsNadE (ATCC; Cat. no. MGC-4508 TT, Lot no. 58263891, cDNA clone MGC:4508 IMAGE: 2966503, GenBank reference AAH03638.1, amino acid residues 1-706) was cloned into pUC19 between the HindIII and EcoRI-restriction sites. The *hsNadE* gene was subcloned into pI-Insect SUMOstar intracellular vector (LifeSensors) using the purified *hsNadE* gene derived from HindIII and EcoRI double digested pUC19*-hsNadE* as a PCR template (see Supplementary Table 3 for primers’ sequences). Phusion DNA polymerase (BioLabs) was used to amplify *hsNadE* using the manufacturer’s protocol. The PCR product was treated with T4 DNA polymerase (BioLab) to increase the efficiency of generating 5′ overhangs for the insert and was then purified by agarose gel. The purified insert was annealed to a linear pI-SUMOstar vector with a ratio of 2.5 μL vector to 2 μL insert in a thermocycler according to manufacturer’s specifications and transformed into GeneHogs *E. coli* cells (Invitrogen). DNA sequencing confirmed the correct construction of pISUMOstar-*hsNadE*. The recombinant bacmid was obtained by the transformation of 1 ng pISUMOstar-*hsNadE* into DH10Bac competent cells (Invitrogen), which was selected using the blue/white screening and was cultured for bacmid extraction.

### Expression of hsNadE in Sf9 insect cells

The hsNadE protein was expressed as an intracellular SUMOstar fusion protein with N-terminal His_6_-tag in Sf9 insect cells. A culture of Sf9 insect cells (Invitrogen) was propagated in Sf9-900 II SFM media (Invitrogen). The confluent monolayer of cells grown in a T75-flask were sub-cultured to a 25-mL shake flask, which were then incubated at 27 °C with constant shaking at 120 rpm until the cells doubled every 18–24 h and were 95% viable. The Sf9 cells were transfected using Cellfectin II reagent (Invitrogen) in a six-well plate following the manufacturer’s protocol. A total 2 μg of bacmid were used for 9 × 10^5^ cells and the infected cells were collected at 72 h post-transfection. The first viral stock was then used to propagate a higher titer viral stock. For protein expression, the cells were infected by recombinant baculovirus with an MOI of ~1 and cultured in a 250-mL shake flask. The cell culture expressing hsNadE was incubated at 27 °C for 72 h at 120 rpm, harvested by centrifugation at 500×*g* for 5 min at room temperature and stored at −80 °C until purification.

### Protein purification of hsNadE

Five to seven milligrams of frozen cells were resuspended in 35 mL lysis buffer containing 50 mM Tris–HCl pH 8.0, 150 mM NaCl, 20% glycerol, 1 mM DTT, 1 mM PMSF, and 300 µl protease inhibitor cocktail (Sigma). Cells were homogenized through a French press cell for four times at 1200 psi and the crude lysate was centrifuged for 1 h at 20,000 × *g*. The soluble lysate was loaded onto a Ni-NTA column equilibrated with buffer A (50 mM Tris–HCl pH 8.0, 300 mM NaCl, 20% glycerol, 20 mM imidazole, 1 mM DTT). The column was washed with 10 CV of buffer A followed by 50 mM imidazole for 10 CV to eliminate non-specific-binding proteins. Highly pure protein was eluted at 200 mM imidazole and dialyzed against 20 mM Tris–HCl pH 8.0, 150 mM NaCl, 10% glycerol, 2 mM DTT. The SUMO fusion tag was cleaved from the protein by adding SUMOstar protease (LifeSensors) to the dialysis bag at a concentration of 1 unit protease per 30 μg of protein for overnight dialysis at 4 °C. Non-fusion protein, free-fusion tag, and un-cleaved protein were separated with the Ni-NTA column re-equilibrated with buffer A. Non-fusion hsNadE was collected and concentrated using an Amicon YM-30 centricon (Millipore) to ~4 mg/mL. The concentrated protein was further purified by Sepharose CL-6B gel filtration (G.E. Healthcare) equilibrated with 20 mM Tris–HCl pH 7.5, 15% glycerol, 1 mM DTT. Homogenous protein with >95% purity, as determined by SDS–PAGE, was obtained in a total yield of 13 mg/L Sf9 insect cell culture based on the protein extinction coefficient *ε*_280_ = 1.39 (±0.02) mL mg^−1^ cm^−1^.

### Cloning, expression, and protein purification of tbNadE

The cloning, expression, and purification methods of tbNadE are described in the Supplementary Methods.

### Steady-state kinetic assays for NAD^+^ synthetase

The synthetase and glutaminase activities were measured by discontinuous assays of alcohol dehydrogenase (NAD^+^ assay) and glutamate dehydrogenase (Glu assay)^22,23^. Briefly, steady-state kinetic assays of hsNadE were performed at 37 °C in 150 mM Tris–HCl pH 8.0, 10 mM MgCl_2_, and 1 mM DTT at a constant ionic strength of 0.1 M adjusted with KCl in the Gln and for the ammonia reactions. Between five to eight different concentrations for the varied substrate (in a similar range as with tbNadE) were analyzed with saturating concentrations of the other two substrates. Saturating concentrations of ATP, NaAD^+^, L-glutamine (glutamine-dependent assay), and NH_4_Cl (ammonia-dependent assay) were, 4, 5, 100, and 200 mM, respectively. The steady-state rate constants were obtained by fitting the kinetic data to the Michaelis–Menten equation using Prism 5 (GraphPad Software). The glutaminase activity was used to determine the activation effect on the glutaminase domain in both hsNadE and tbNadE. For hsNadE, the Glu assay was performed in the presence of ATP (4 mM), NaAD^+^ (5 mM), and L-glutamine (1.2–75 mM), and also with AMP (5 mM), PPi (2.5 mM), NAD^+^ (4 mM), and L-glutamine (50 mM). For tbNadE, the glutaminase assay was performed with the SF1 (5 mM) and L-glutamine (20 mM) in the presence and absence of PPi (2.5 mM).

Stoichiometric data were obtained to calculate the channel efficiency based on the ratio of NAD^+^ produced from the synthetase activity to glutamate generated from the glutaminase activity measured as described above. Product formation of NAD^+^ and glutamate were determined by NAD^+^ assay and Glu assay, respectively, in the presence of L-glutamine (2.4, 5, 24, and 75 mM) at saturating concentrations of NaAD^+^ (5 mM) and ATP (4 mM).

### Synthesis of SFI

The synthetic procedure to prepare SFI is described in the Supplementary Methods.

### Crystallization

The hsNadE protein at 9 mg/mL was co-crystallized with NaAD^+^ (4 mM), AMP (10 mM), PPi (10 mM), and MgCl_2_ (40 mM). Crystallization conditions of this complex were screened with six screening kits; Index (Hampton Research), Cryo suite (QIAGEN), and Wizard I–IV (Emerald Biosystems) using a Phoenix screening robot station (Art Robbins Instruments). Optimization by systematic trials was performed using the hanging drop vapor-diffusion method at 15 °C. Needle-like crystals appeared after 1 week with an average size of 60 × 150 × 30 μm in 0.8 M (NH_4_)_2_SO_4_, 30% MPD, 15% glycerol, 85 mM HEPES (pH 7.5) with a ratio of 2 μL protein to 1 μL precipitant. For the tbNadE crystal in complex with SFI, the C176A mutant enzyme was co-crystallized with 5 mM SFI, 4 mM PPi, and 20 mM L-glutamine. The thin plate-like crystal was grown to the maximal size of 100 × 200 × 10 μm after 3 months in 1.6 M ammonium citrate tribasic (pH 8.0), 10% glycerol, and 120 mM MgCl_2_.

### Data collection and processing

For the hsNadE crystal, 30% MPD and 15% glycerol included in crystallization condition were used as cryoprotectants, while 15% glycerol was used for the tbNadE-SFI crystal^22^. Diffraction data were collected from the hsNadE crystal to 2.84 Å resolution at BL7-1 at SSRL on a Q315R detector. Two data sets of low and high resolution containing 300 images with 0.5 oscillations were collected from a single crystal. Data sets were combined for data processing and scaling using XDS^44^. The crystal belonged to space group*I*222, with two molecules per asymmetric unit. The calculated Matthew’s coefficient (*V*_m_) was 3.54 Å/Da, and the solvent content was 65.3%. The tbNadE-SFI crystal diffracted to 3.14 Å resolution at BL9-2 at SSRL on a MAR325 detector. A total of 772 images with an oscillation range of 0.35° were collected and processed by using DENZO and SCALEPACK from HKL2000 program package^45^. The crystal belonged to space group *P*4_1_2_1_2, with four molecules per asymmetric units as previously reported^22^.

### Structure determination

The hsNadE structure was determined by molecular replacement by using the program AutoMR wizard in PHENIX^46^ with glutamine-dependent NAD^+^ synthethase from *Cytophaga hutchinsonii* (PDB code 3ILV, 23% identity) as a search model. The 3ILV structure was used rather than the tbNadE structure (PDB code 3DLA, 18% identity) because it shares higher sequence identity. The initial model was derived from the AutoBuild wizard in PHENIX^46^ consisting of 1053 of the expected 1412 residues (molecules A and B) and resulted in *R*_free_ and *R*_factor_ of 35.0% and 40.0%. Manual model rebuilding was performed iteratively in COOT^47^, and refined in Phenix.refine^46^. Clear density maps were observed for the main chains of α-helices and β-strands from the first model. Additional main chains were built into the structure using the “place helix/strand here” feature in COOT. The residues exposed to solvent and the two flexible loops in the synthetase domain were added after cycles of refinement when the electron density map was improved. After the refinement converged, further refinement was carried out in BUSTER^48^ to 2.84 Å resolution. The ligands were added based on the composite omit map calculated by CNS^49^ and water molecules were checked for potential interactions with protein based on *F*_O_−*F*_C_ (>3*σ*) electron density map. The last cycle of refinement was done in Phenix.refine, with *R*_work_ and *R*_free_ of 17.9%, 21.7%. The final hsNadE structure consists of 1390 residues, with two molecules of NaAD^+^, AMP, PPi, and Mg^2+^ as co-crystallized ligands. The structure of tbNadE was also solved by molecular replacement using the tbNadE structure bound to DON/NaAD^+^ (3DLA) as a search model^22^. The structure was refined to 3.14 Å resolution by Phenix.refine during cycles of model building in COOT. The 3D structure coordinates of the SFI ligand was derived from the 3D structure generator CORINA^50^, available from Molecular Networks (http://www.molecular-networks.com), and its dictionary was generated by XPLO2D^51^. The ligand was added at the last few cycles of refinement based on the composite omit map calculated by CNS. The final model includes a tetramer consisting 2655 of the expected 2716 residues, with eight molecules of SFI and 3 glutamines. The residues for the P1 loop (403–406), P2 loop (544–553), and region 2 (442–451) were not present in some subunits of the tbNadE in complex with glutamine, SFI, and PPi complex. Both structures were validated in Molprobity^52^ and Quality Control Check from JCSG. The molecular tunnel was calculated by CAVER program^53^.

### Reporting summary

Further information on research design is available in the Nature Research Reporting Summary linked to this article.

## Supplementary information

Supplementary Information

Reporting Summary

## Data Availability

The protein coordinates and structure factors of hsNadE in complex with NaAD^+^, AMP, and MgPPi and tbNadE in complex with glutamine, SFI, and PPi have been deposited with PDB code 6OFB and 6OFC, respectively. The source data underlying Tables 1 and 2, and Supplementary Table 1 are provided as a Source Data file. Other data are available from the corresponding author upon reasonable request.

## Source Data

Source Data
