# Supplementary material for: Different ways to transport ammonia in human and Mycobacterium tuberculosis NAD+ synthetases
Source: Nat Commun. 2020 Jan 7;11:16. doi: 10.1038/s41467-019-13845-4 (PMC6946656; doi:10.1038/s41467-019-13845-4)
Supplement: Supplementary file 1 — Supplementary Information [file 41467_2019_13845_MOESM1_ESM.pdf]

SUPPLEMENTARY INFORMATION

Different ways to transport ammonia in human and *Mycobacterium tuberculosis* NAD$^+$ synthetases

W. Chuenchor *et al*.

Supplementary Methods

Cloning, expression and purification of tbNadE

tbNadE was cloned, expressed and purified as perviosusly described[1]. Briefly, the gene encoding NAD^+ synthetase from *M. tuberculosis* genomic DNA was amplified and cloned it into pSMT3 vector (see Supplementary Table 3 for primers' sequences). The protein was expressed in *Escherichia coli* BL21(DE3) in LB medium containing kanamycin (50 mg/ml/l) and induced with 0.2% (w/v) lactose at 18°C for 48 h. Frozen cells were suspended to 0.1 g ml–1 in ice-cold lysis buffer (50 mM Tris, pH 8.0, 150 mM NaCl, 1 mM benzamidine, 1 mM PMSF, 1 mM DTT, 20% (v/v) glycerol) and lysed by French press at 12,000 psi. Cell debris were removed by centrifugation and the protein purified on a nickel–nitrilotriacetic acid (NiNTA) agarose resin (Qiagen) with a linear gradient of 20–120 mM imidazole in 50 mM Tris, pH 8.0, 300 mM NaCl, 1 mM DTT, 20% (v/v) glycerol. The SUMO tag was cleaved by dialysis (50 mM Tris, pH 8.0, 1 mM DTT, 350 mM NaCl, 30% (v/v) glycerol) with the protease Ulp1 at 4°C for 3 h. NAD^+ synthetase was then dialyzed in 20 mM Tris, pH 7.5, 1 mM DTT, 30% (v/v) glycerol and ran on a Sepharose CL-6B column (Amersham Biosciences) with 20 mM Tris, pH 7.5, 1 mM DTT and 15% (v/v) glycerol.

Synthesis of sulfonamide derivative 1 (SFI)

5'-*O*-(*tert*-Butyldimethylsilyl)-N^6-*tert*-butoxycarbonyl-2',3'-*O*-isopropylideneadenosine (1)

TBDMSCl (56.7 mmol, 8.55 g) was added to a solution of 2',3'-*O*-Isopropylideneadenosine (16.2 mmol, 5.00 g) and imidazole (24.3 mmol, 1.65 g) in CH_2Cl_2 (120 ml) at 0 °C. The resulting solution was stirred at r.t. for 16 h, then filtered and the filtrate was concentrated under reduced pressure. The crude solid was directly used for the next step.

NaH (60% dispersion in mineral oil, 17.8 mmol, 0.71 g) was slowly added to a solution of the above solid in THF (200 ml) and DMF (50 ml) at 0 °C. After 30 min at 0 °C, a solution of Boc_2O (16.2 mmol, 3.54 g) in

THF (10 ml) was added via cannula followed by another addition after 30 min at 0 °C of Boc_2O (8.1 mmol, 1.77 g) in THF (10 ml). The reaction mixture was allowed to reach r.t. and stirred for 16 h. The solution was diluted with CH_2Cl_2 and washed with saturated aq. $NaHCO_3$. The organic phase was washed with H_2O and brine, dried ($MgSO_4$), and concentrated. Purification by flash chromatography (EtOAc/hexanes = 2/1) afforded the compound **1** (2.1 g, 25%): ^{1}H NMR (300 MHz, $CDCl_3$) □ (ppm) -0.0033 (m, 6H), 0.82 (s, 9H), 1.41 (s, 3H), 1.56 (s, 9H), 1.64 (s, 3H), 3.78 (dd, 1H, *J* = 11 Hz, 2 Hz), 3.90 (dd, 1H, *J* = 11 Hz, 2 Hz), 4.47 (m, 1H), 4.94 (dd, 1H, *J* = 6 Hz, 2Hz), 5. 26 (dd, 1H, *J* = 6 Hz, 2Hz), 6.20 (d, 1H, *J* = 2.7 Hz), 8.19 (s, 1H), 8.79 (s, 1H).

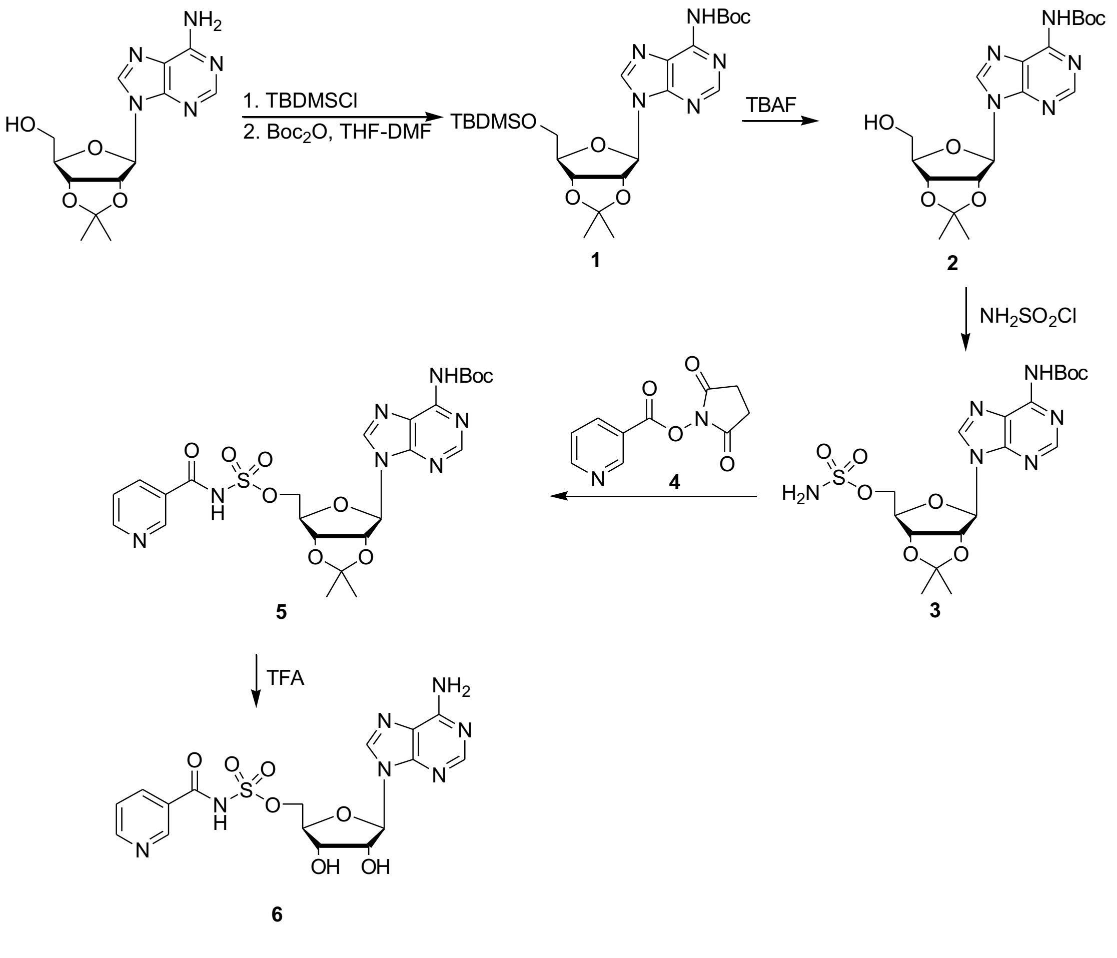

N^6-*tert*-Butoxycarbonyl-2',3'-*O*-isopropylideneadenosine (2)

TBAF (1M solution in THF, 6.92 mmol, 6.92 ml) was added to a solution of **1** (4.61 mmol, 2.40 g) in THF (50 ml) at rt. After 4h at r.t., the reaction mixture was concentrated under reduced pressure. Purification by flash chromatography (EtOAc/hexanes = 5/1) afforded the compound **2** (1.29 g, 69%): ^{1}H NMR (300 MHz, $CDCl_3$) □ (ppm) 1.37 (s, 3H), 1.55 (m, 12H), 1.64 (s, 3H), 3.79 (m, 1H), 3.96 (m, 1H), 4.12 (m, 1H), 4.55 (s, 1H), 5.11 (m, 1H), 5.19 (m, 1H), 5.90 (m, 1H), 8.20 (s, 1H), 8.72 (s, 1H).

5'-*O*-(Sulfamoyl)-N^6-*tert*-butoxycarbonyl-2',3'-*O*-isopropylideneadenosine (3)[2,3]

NaH (60% dispersion in mineral oil, 6.63 mmol, 0.26 g) was added to a solution of **2** (4.42 mmol, 1.80 g) in DME (100 ml) at 0 °C. After 30 min at 0 °C, a solution of sulfamoyl chloride[3] (1.5 g) in DME (30 ml) was added over 20 min. After warming to r.t. and stirred for 16h, the reaction mixture was quenched with MeOH at 0 °C and concentrated under reduced pressure. Purification by flash chromatography (EtOAc/hexanes = 5/1) afforded the compound **3** (0.87 g, 41%): ^{1}H NMR (300 MHz, $CDCl_3$) □ (ppm) 1.37 (s, 3H), 1.52 (s, 9H), 1.60 (s, 3H), 4.36 (m, 2H), 4.57 (m, 1H), 5.09 (m, 1H), 5.38 (m, 1H), 6.06 (br s, 1H), 6.21 (s, 1H), 8.17 (s, 1H), 8.70 (s, 1H); MS (ES+) [M+H] 487.2. CAS registry number 873556-35-5.

5'-*O*-[*N*-(Nicotinyl)sulfamoyl]-N^6-*tert*-butoxycarbonyl-2',3'-*O*-isopropylideneadenosine (5)

N-Hydroxysuccinimdyl nicotinate**4** (2.70 mmol, 594 mg) and Cs_2CO_3 (2.70 mmol, 521 mg) was added to a solution of **3** (1.80 mmol, 876 mg) in DMF (20 ml) at -20 °C. The reaction mixture was warmed to r.t. and stirred for 16h. The reaction mixture was concentrated under reduced pressure and the residue was treated with EtOAc (50 ml) and filtered. The crude solids were washed with additional EtOAc (100 ml) and the combined filtrate was concentrated. Purification by flash chromatography (EtOAc/MeOH/Et_3N = 95/5/1) afforded the compound **5** (910 mg, 86%): ^{1}H NMR (300 MHz, CD_3OD) □ (ppm) 1.58 (s, 12H), 4.32 (m,

2H), 4.60 (m, 1H), 5.16 (m, 1H), 5.42 (m, 1H), 6.29 (d, 1H, *J* = 2.7 Hz), 7.42 (m, 1H), 8.30 (m, 1H), 8.55 (m, 2H), 8.63 (s, 1H), 9.09 (s, 1H); MS (ES+) [M+H] 592.2.

5'-*O*-[*N*-(Nicotinyl)sulfamoyl]adenosine triethylammonium salt (6)

80% aq. TFA (30 ml) was added to **5** (1.35 mmol, 800 mg) at 0 °C then the reaction mixture was slowly warmed to r.t. After 2h at r.t., the reaction mixture was concentrated under reduced pressure. Purification by flash chromatography (EtOAc/MeOH/Et_3N = 75/25/1) afforded the compound **6** (524 mg, 70%): ^1H NMR (300 MHz, CD_3OD) □ (ppm) 1.27 (m, 9H), 3.20 (m, 6H), 4.32-4.41 (m, 4H), 4.69 (m, 1H), 6.08 (d, 1H, *J* = 5.8 Hz), 7.42 (m, 1H), 8.14 (s, 1H), 8.38 (m, 1H), 8.53 (m, 2H), 9.13 (s, 1H); MS (ES+) [M+H] 452.2.

References

1. LaRonde-LeBalnc N, *et al*. Regulation of active site coupling in glutamine dependent NAD^+ synthetase. *Nature Structural & Molecular Biology* **16**, 421-9 (2009).
2. Somu RV, *et al*. Rationally designed nucleoside antibiotics that inhibit siderophore biosynthesis of *Mycobacterium tuberculosis*. *J. Med. Chem.* **49**, 31-34 (2006).
3. Ishikawa F, Kakeya H. Specific enrichment of nonribosomal peptide synthetase module by an affinity probe for adenylation domains. *Bioorganic & Medicinal Chemistry Letters* **24**, 865-869 (2014).

Supplementary Tables

Supplementary Table 1. Stoichiometry analysis for hsNadE catalyzed reaction

Gln concentration	Glu/NAD^+	% Channel Efficiency
2.4	1.38 ± 0.12	72 ± 6
5.0	1.22 ± 0.06	82 ± 4
10.0	1.17 ± 0.11	85 ± 8
24.0	1.39 ± 0.02	72 ± 1
50.0	1.4 ± 0.1	71 ± 6
75.0	1.41 ± 0.04	71 ± 2

Supplementary Table 2. X-ray diffraction data and structure refinement Statistics

Crystal structure	hsNadE (6OFB)	tbNadE (6OFC)
Space group	*I*222	$P4_12_12$
Unit cell *a*, *b*, *c* (Å)	102.3, 198.9, 219.7	179.3, 179.3, 208.2
α, β, γ (°)	90.0, 90.0, 90.0	90.0, 90.0, 90.0
Wavelength (Å)	0.9795	0.9795
Resolution range (Å)	40-2.84 (2.91-2.84)	39.56-3.14 (3.31-3.14)
Total reflections	216,548 (16057)	660,246 (71,651)
Unique reflections	52456 (3857)	58,806 (8,258)
Completeness (%)	99.5 (99.7)	99.0 (96.4)
Redundancy	4.1 (4.2)	11.2 (8.7)
$I/\sigma I$	13.0 (1.3)	12.9 (3.5)
R_{merged}(%)	10.5 (118.3)	18.1 (64.4)
**Refinement**		
Data used in refinement (Å)	37.4-2.84 (2.99-2.84)	36.1-3.14
No. of reflections used in refinement	52445 (4764)	58620 (5514)
No. of reflections for R_{free}	2668 (241)	2962 (260)
R_{work}(%), R_{free} (%)	17.9 / 21.7	18.6 / 23.5
No. of atoms		
Protein	11012	20517
All heteroatom	81	334
Mean *B*-factor ($Å^2$)		
Protein	72.66	51.8
$NaAD^+$ or SFI	167.7[A] / 167.7[B]	78.9[A] / 72.5[B] / 74.8[C] / 75.9[D]
AMP or SFI	155.5[A] / 155.5[B]	68.3[A] / 59.0[B] / 55.4[C] / 69.2[D]
PPi	159.7[A] / 155.8[B]	None[A]/63.4[B]/ None[C]/None[D]
Mg^{2+}	113.6[A] / 107.4[B]	None
Glutamine	None	None[A] / 57.3[B] / 60.0[C] / 76.2[D]
Glycerol	None	48.5
Cl^-	81.2	67.1
Solvent	53.95	45.0
RMSD from ideality		
Bond lengths (Å)	0.011	0.007
Bond angles (deg.)	1.361	0.947
**Validation**		
Ramachandran analysis		
Favored (%)	94.4	95.0
Allowed (%)	99.6	100
Outliers (%)	0.4	0
Rotamer outliers (%)	6.2	2.3
Clashscore	8.9	8.8

Supplementary Table 3. Primer sequences used for cloning of hsNadE and tbNadE

Name	Sequence	Length (nt)	Tm (°C)
hsNadE_F	5'-CAC AGA GAA CAG ATT GGA GGT ATG GGC CGG AAG GTG ACC GTG-3'	42	82
hsNadE_R	5'-GTA CCG CGG CCG CTC TAG TCA GTC CAC GCC GTC CAG GGA-3'	39	88
tbNadE_F	5'-GGC GGC GGA TCC ATG AAC TTT TAC TCC GCC-3'	30	52
tbNadE_R	5'-GGT GGT AAG CTT CTA GCC CTT GGG CAC C-3'	28	54

Supplementary Figures

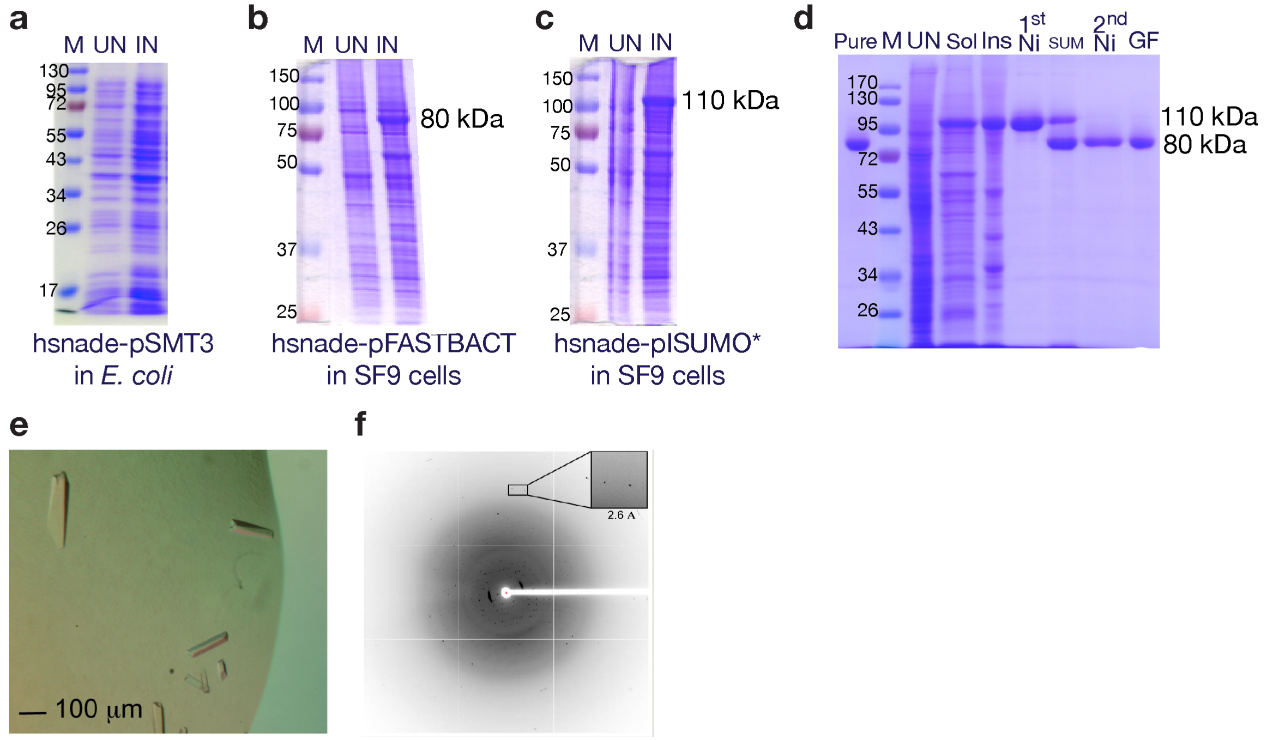

Supplementary Figure 1. Protein expression, purification and crystallization of hsNadE. **A-D.** SDS-PAGE of protein expression level of hsNadE as hsnade-pSMT3 (with SUMO/His$_6$) construct in *E. coli* (A); hsnade-pFASTBACT (His$_6$) construct in Sf9 cells (B); hsnade-pISUMO* (with SUMO*/His$_6$) construct in Sf9 cells (C); and overall SDS-PAGE of hsNadE purification (D). M: protein marker, UN: uninfected cells, IN: infected cells, Sol: soluble protein, Ins: insoluble fraction, 1stNi: 1st Ni column, SUM: proteolysis by SUMO* protease, 2ndNi: 2nd Ni column, GF: Sepharose CL-6B gel filtration. **E.** crystals of hsNadE in complex with NaAD$^+$, AMP, and MgPPi. Needle-like crystals (60 × 150 × 30 µm) from optimization plate grew in 0.8 M

$(NH_4)_2SO_4$, 30% MPD, 15% glycerol, 85 mM HEPES (pH 7.5) after 7 days growth. **F.** These crystals diffracted at SSRL synchrotron between 2.69-3.30 angstrom resolutions.

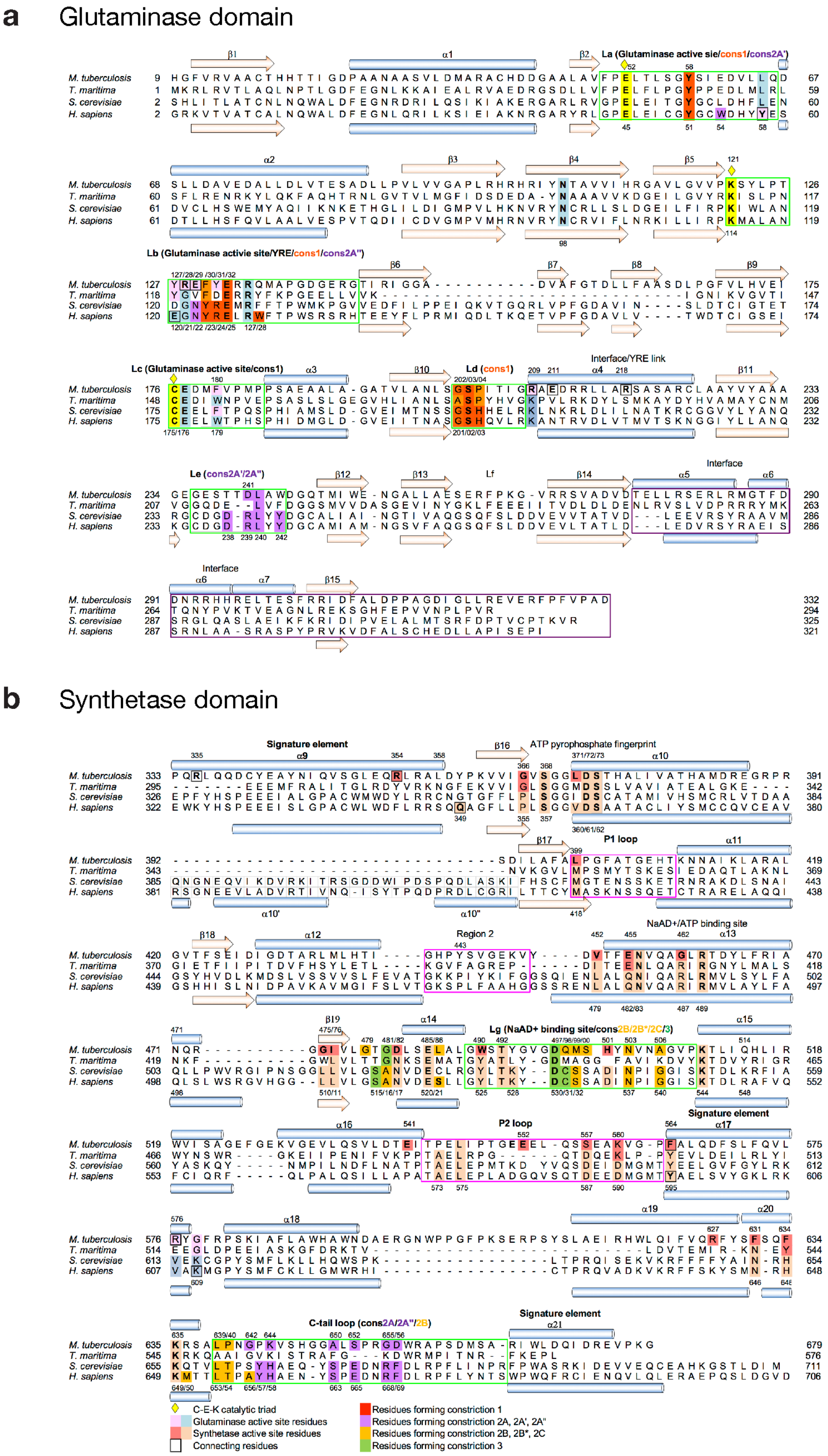
a Glutaminase domain
M. tuberculosis
T. maritima
S. cerevisiae
H. sapiens
La (Glutaminase active site/cons1/cons2A')
Lb (Glutaminase activie site/YRE/cons1/cons2A")
Lc (Glutaminase active site/cons1)
Ld (cons1)
Interface/YRE link
Le (cons2A'/2A")
Lf
Interface
b Synthetase domain
Signature element
ATP pyrophosphate fingerprint
P1 loop
Region 2
NaAD+/ATP binding site
Lg (NaAD+ binding site/cons2B/2B*/2C/3)
P2 loop
C-tail loop (cons2A/2A"/2B)
C-E-K catalytic triad
Glutaminase active site residues
Synthetase active site residues
Connecting residues
Residues forming constriction 1
Residues forming constriction 2A, 2A', 2A"
Residues forming constriction 2B, 2B*, 2C
Residues forming constriction 3

Supplementary Figure 2. Structure-based alignment of ^{gln}NAD^{+} synthetases from prokaryotes and eukaryotes. **A-B.** The two glutaminase (A) and synthetase (B) domains are shown separately. The secondary structure (β-sheet; β, α-helix; α, and loop; L) of tbNadE (3DLA) and hsNadE (6OFB) are shown above and below the alignment, respectively. Yellow diamond labels the conserved catalytic triad residues.

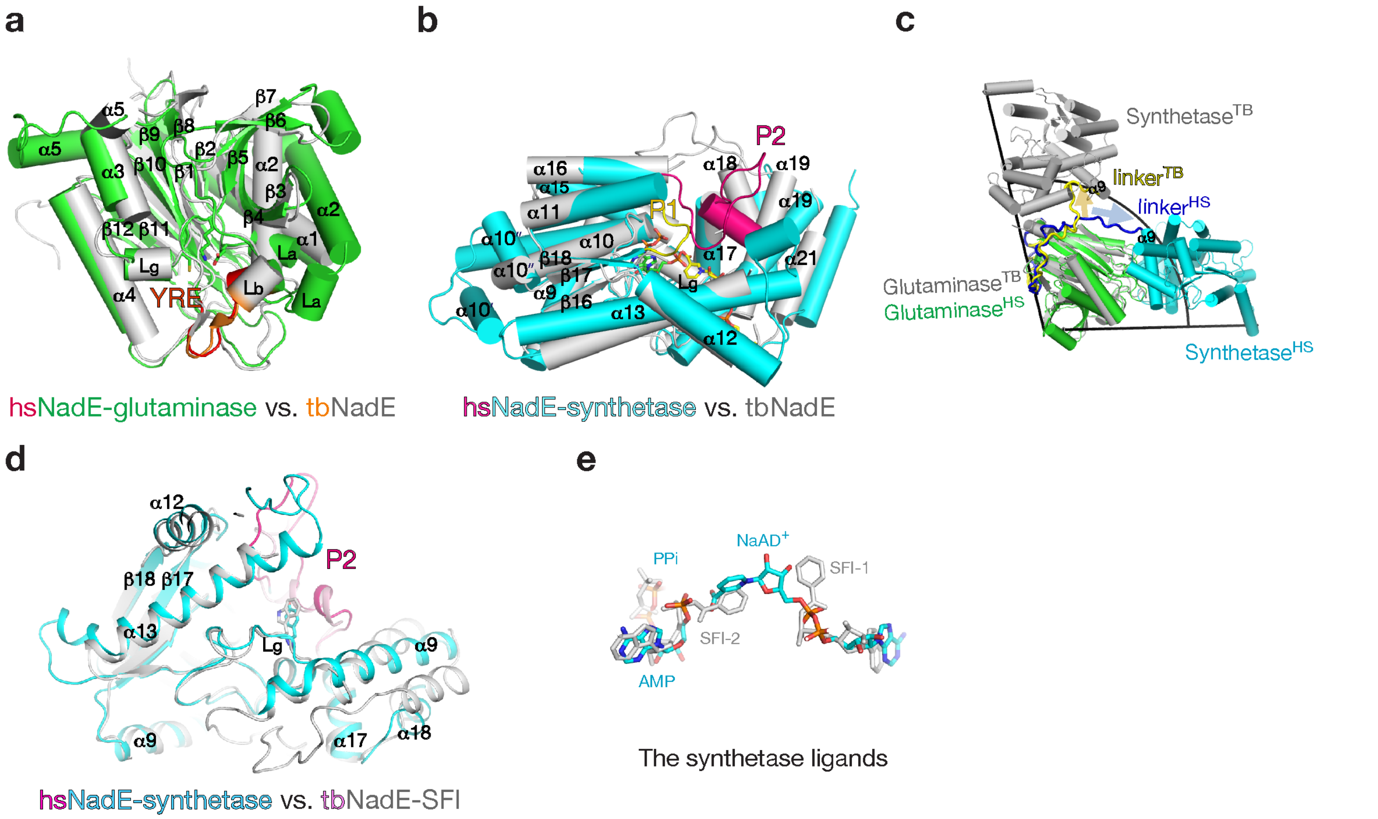

Supplementary Figure 3. Comparison of the glutaminase and synthetase domains of hsNadE and tbNadE. **A-B.** Superimposition of the glutaminase (A) and synthetase domains (B) between the two homologs. **C.** Highlight of the different orientations of the synthetase domain between the two structures. The glutaminase domains alone of hsNadE (green) and tbNadE (grey) are used for superposition. The synthetase domain of hsNadE (cyan) translates by 103° in respect to the synthetase domain in tbNadE (grey). The linker between the glutaminase and synthetase domains places the synthetase domains in a different location as shown in dark blue and yellow for hsNadE and tbNadE. The direction of the synthetase domain from the glutaminase domain is indicated by arrows. **D-E.** Truncation elements of the synthetase domain used for structural comparison between the two homologs to improve the structural overlay of ligands bound in the synthetase active site.

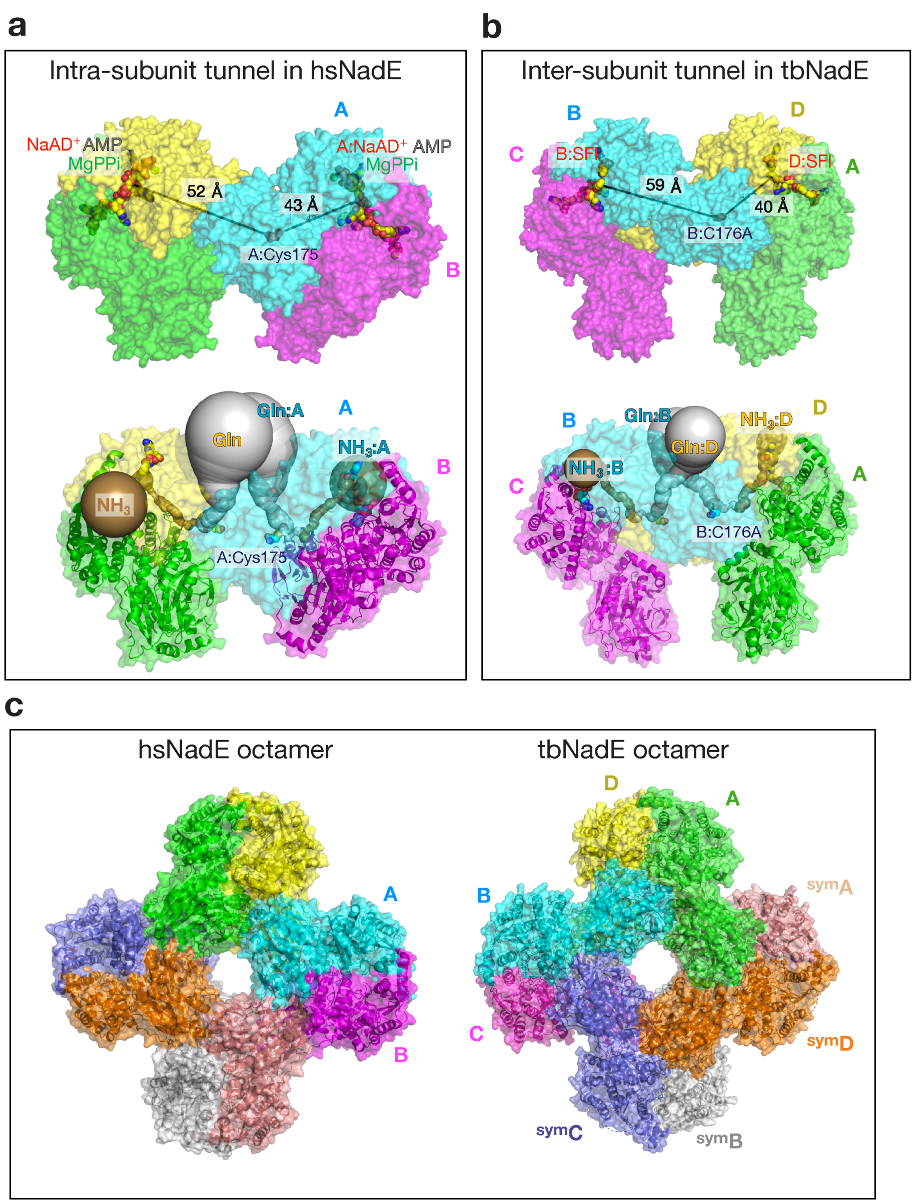

Supplementary Figure 4. Octameric assembly, intra- and inter-subunit tunnels in hsNadE and tbNadE. **A-B.** Distance between the glutaminase and synthetase active sites within one subunit and between two adjacent subunits in hsNadE (B) and tbNadE (C). Intra-subunit tunnels in hsNadE and inter-subunit tunnels in tbNadE are calculated by the Caver program. Three residues of Trp54^{HS}, Tyr51^{HS}, and Leu240^{HS} in hsNadE and Glu61^{TB}, Tyr58^{TB}, and Leu242^{TB} in tbNadE were used as a crisscross for tunnel calculation. **C.** Comparison of the octameric hsNadE and tbNadE generated by symmetry-related molecules from crystal structure.

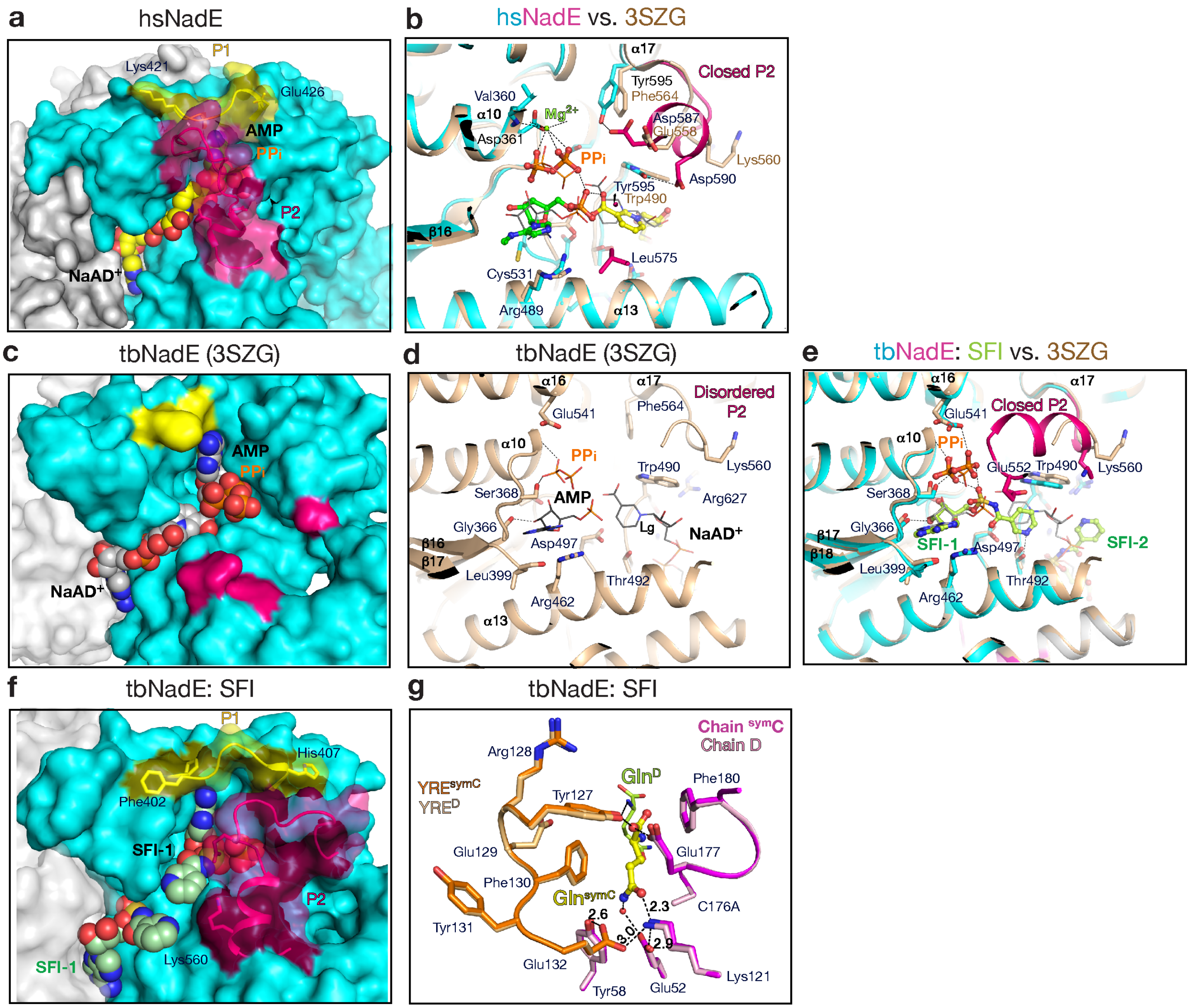

Supplementary Figure 5. Ordering of P2 loop upon binding of the synthetase intermediate analogue. **A.** Surface of the synthetase active site in the closed P loop of hsNadE. **B.** Overlay of the synthetase active sites in the open tbNadE (PDB 3SZG) and closed hsNadE. **C-D.** The open P2 loop structure reported previously in tbNadE with $NaAD^+$, AMP, and PPi bound (PDB 3SZG). **E.** Overlay of the synthetase active sites in the open and closed P2 loop conformations; 3SZG and tbNadE-SFI. **F.** Surface of the synthetase active site in the closed P loops of tbNadE-SFI. G. Overlay chain D^{glu} and $^{sym}C^{glu}$ of the glutaminase active site upon binding of the SFI, PPi in tbNadE-SFI complex.

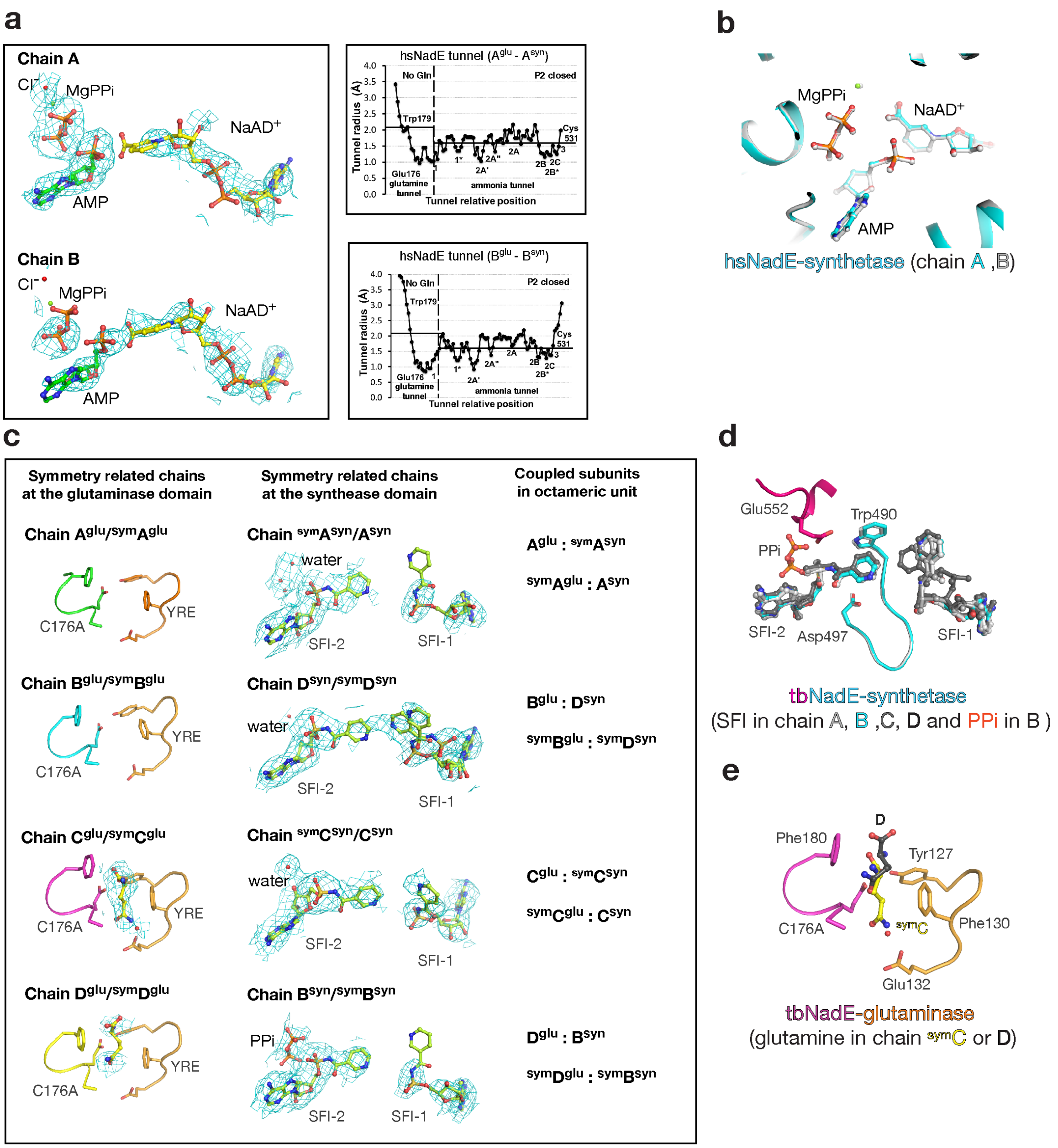

Supplementary Figure 6. Comparison of ligands bound in each coupled subunit in hsNadE and tbNadE complexes. **A.** Ligands bound in each subunit with composite omit map (cyan mesh, $2F_o$-F_c, 0.7σ) calculated by PHENIX[46] and tunnel radii calculated in subunits A and B of hsNadE. **B.** Superimposition of ligands in chain A and B of the hsNadE synthetase active site. **C.** Composite omit map (cyan mesh, $2F_o$-F_c, 0.7σ) of ligands in the tbNadE-SF complex. **D-E.** Superimposition of ligands in different subunit of the tbNadE synthetase active sites (D), and tbNadE glutaminase active site (E).

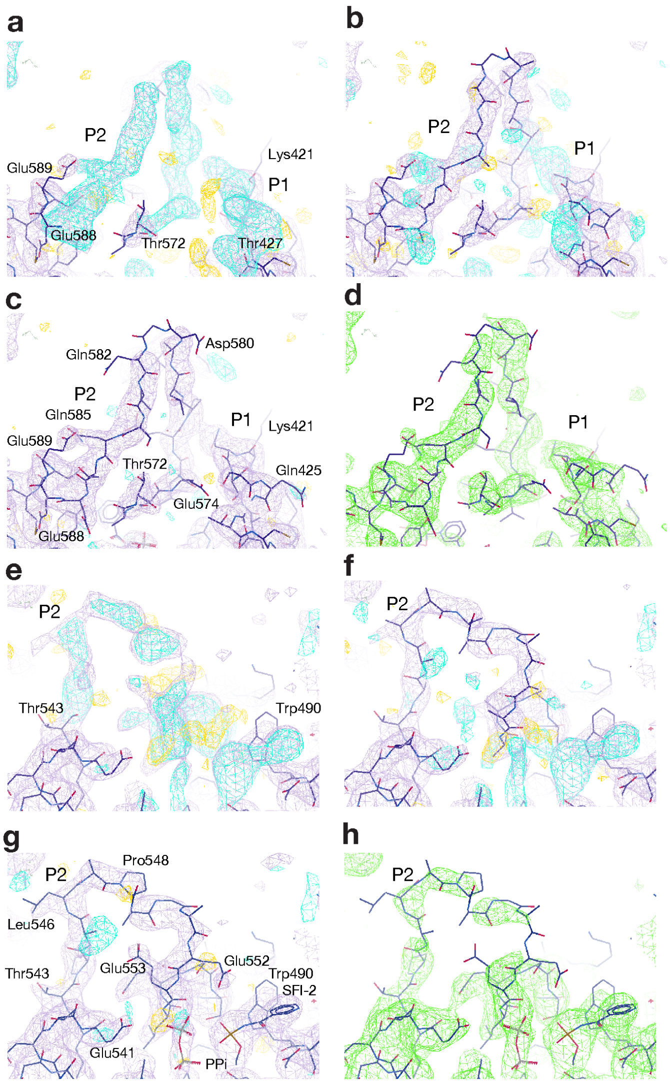

Supplementary Figure 7. Electron density of P2 loop in rebuilding and refinement of hsNadE and tbNadE structures. hsNadE structures are shown in panels A-D and tbNadE in E-H. $2F_o$-F_c, 1σ shown in purple and F_o-F_c, 3σ shown in cyan/orange indicate the electron density of P2 loop when traced for the first time in both complexes (A, E). The main chain of the P2 loop was subsequently built in (B, F) followed by their side chains (C, G). The $2F_o$-F_c composite omit map: 1σ is shown in green in the two final structures (D, H).

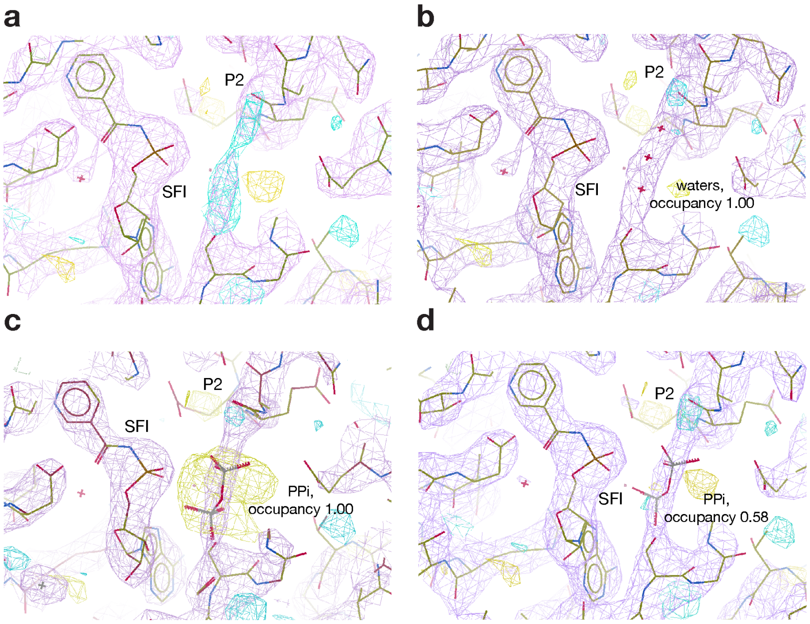

Supplementary Figure 8. Partial occupancy of PPi molecule in subunit B of tbNadE-SFI complex. **A.** Positive map (blue mesh, F_o-F_c, 3.0σ) observed close to SFI. **B.** Refinement after adding three water molecules at occupancy of 1.0. **C.** Observation of negative density of PPi at occupancy of 1.0 (green map, F_o-F_c, 3.0σ). **D.** Occupancy of PPi was refined to 0.58.

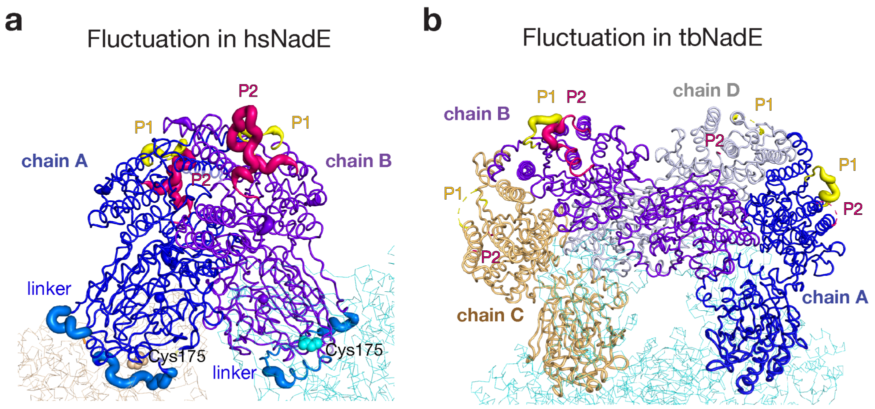

Supplementary Figure 9. Magnitude of the atomic fluctuation in hsNadE and tbNadE structures. **A-B.** The absolute atomic motion in hsNadE and tbNadE complexes was analysed by DynaMut [31]. The magnitude of the fluctuation is shown by thin to thick tube and colored in the same was as in the diagram in Figure 2.

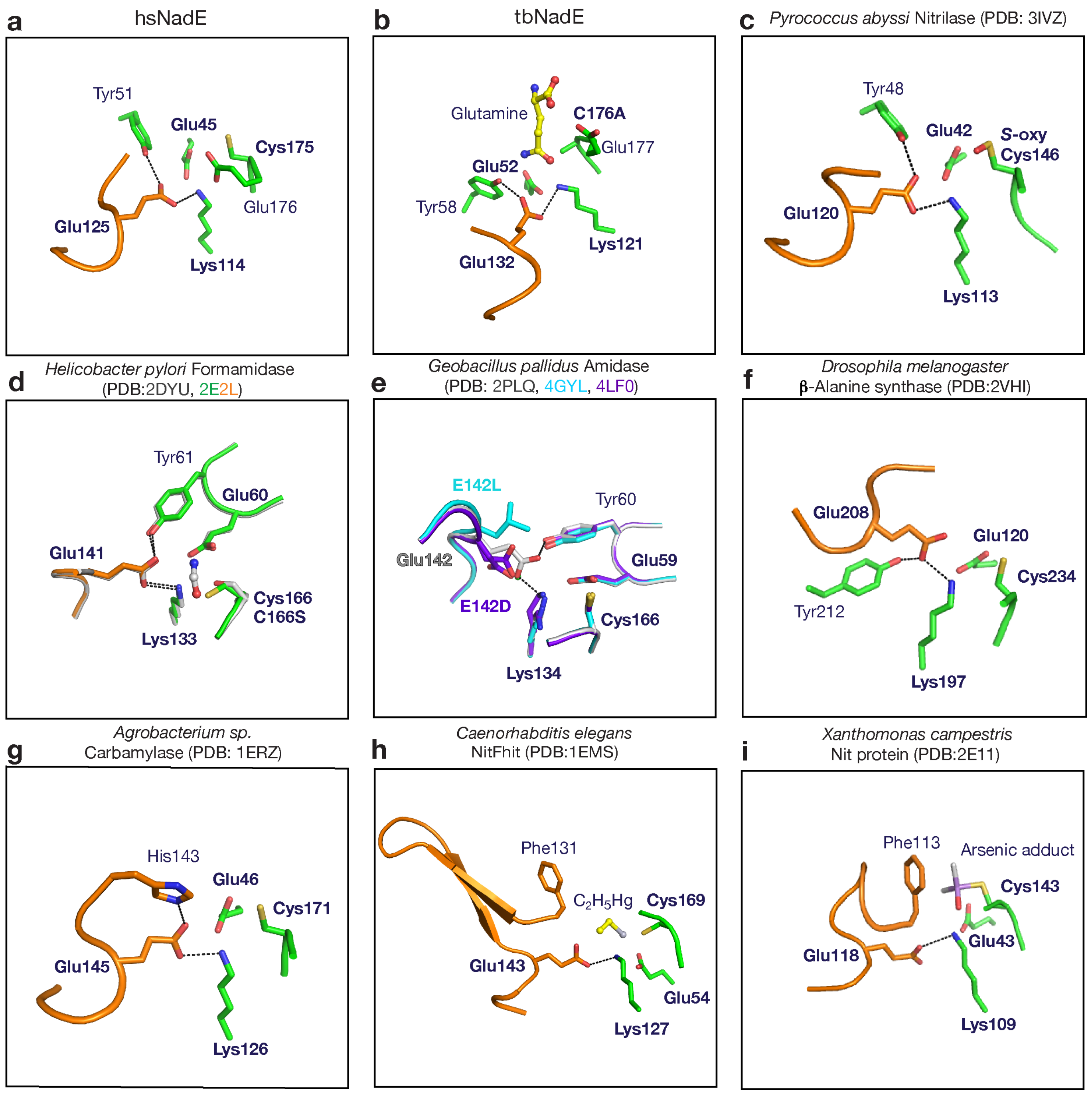

Supplementary Figure 10. Structural comparison of C-E-K triad of the glutaminase active site. The comparison is shown among six subfamilies of the nitrilase superfamily including nitrilase from *Pyrococcus abyssi* [38] (PDB code 3IVZ), formamidase (AmiF) from *Helicobacter pylori* [37] (PDB code 2DYU, 2E2L), amidase from *Geobacillus pallidus* [36](PDB code 4GYL, 4LF0, and 2PLQ), β-alanine synthase [39] (PDB code

2VHI), N-carbamyl-D-amino acid amidohydrolase (DCase) from *Agrobacterium sp*. [40] (PDB 1ERZ), and NitFhit from *Caenorhabditis elegans* [41] (PDB code 1EMS). **A-I.** The strictly conserved glutamate located on the loop (orange) adjacent to the C-E-K triad (green sticks marked in bold labels) stabilizes the side chain of the active site lysine on one side and interacts with tyrosine (A-F) or histidine (G) on the other side. Tyrosine/histidine stabilizing the glutamate located on the loop is not observed in NitFhit and Nit protein but the hydrogen bonding between glutamate and catalytic lysine remains conserved (H-I).

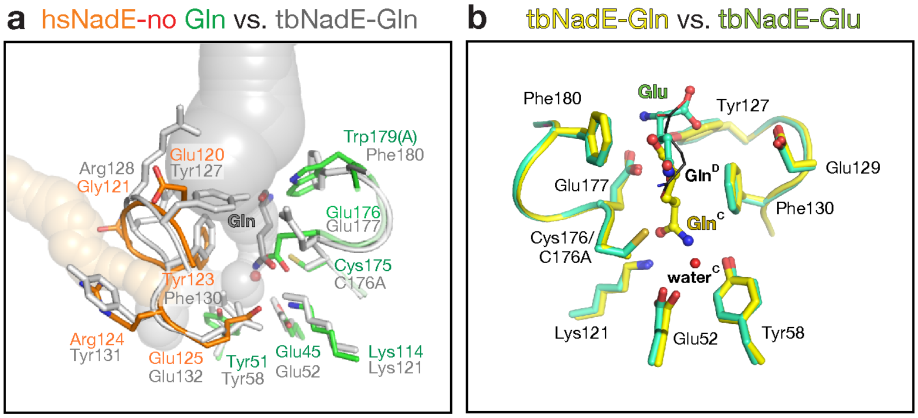

Supplementary Figure 11. Superposition of the glutaminase active sites and YRE loops. **A.** Superposition of the glutaminase active sites and YRE loops of hsNadE vs. tbNadE in the complex with glutamine, SFI, and PPi. **B.** Comparison of glutamine position in different subunits (chains B, C, and D) and glutamate in tbNadE- product complex (PDB code 3SYT).
