# Supplementary material for: Different ways to transport ammonia in human and Mycobacterium tuberculosis NAD+ synthetases
Source: Nat Commun. 2020 Jan 7;11:16. doi: 10.1038/s41467-019-13845-4 (PMC6946656; doi:10.1038/s41467-019-13845-4)
Supplement: Supplementary file 2 — Reporting Summary [file 41467_2019_13845_MOESM2_ESM.pdf]

nature research

Corresponding author(s): *Double-blind peer review submissions: write DBPR and your manuscript number here instead of author names.*

Last updated by author(s): 06/27/2019

Reporting Summary

Nature Research wishes to improve the reproducibility of the work that we publish. This form provides structure for consistency and transparency in reporting. For further information on Nature Research policies, see Authors & Referees and the Editorial Policy Checklist.

Statistics

For all statistical analyses, confirm that the following items are present in the figure legend, table legend, main text, or Methods section.

n/a	Confirmed	
☐	☒	The exact sample size (*n*) for each experimental group/condition, given as a discrete number and unit of measurement
☐	☒	A statement on whether measurements were taken from distinct samples or whether the same sample was measured repeatedly
☒	☐	The statistical test(s) used AND whether they are one- or two-sided<br>*Only common tests should be described solely by name; describe more complex techniques in the Methods section.*
☒	☐	A description of all covariates tested
☒	☐	A description of any assumptions or corrections, such as tests of normality and adjustment for multiple comparisons
☒	☐	A full description of the statistical parameters including central tendency (e.g. means) or other basic estimates (e.g. regression coefficient) AND variation (e.g. standard deviation) or associated estimates of uncertainty (e.g. confidence intervals)
☒	☐	For null hypothesis testing, the test statistic (e.g. *F*, *t*, *r*) with confidence intervals, effect sizes, degrees of freedom and *P* value noted<br>*Give P values as exact values whenever suitable.*
☒	☐	For Bayesian analysis, information on the choice of priors and Markov chain Monte Carlo settings
☒	☐	For hierarchical and complex designs, identification of the appropriate level for tests and full reporting of outcomes
☒	☐	Estimates of effect sizes (e.g. Cohen's *d*, Pearson's *r*), indicating how they were calculated

Our web collection on statistics for biologists contains articles on many of the points above.

Software and code

Policy information about availability of computer code

Data collection	The Stanford Automated Mounting system, Web-Ice, NoMachine.
Data analysis	Data reduction: XDS, SCALA, DENZO, SCALEPACK, HKL2000; Refinement: PHENIX 1.1_743, PHENIX 1.12_2829, PHENIX 1.16_3549, BUSTER; Structure solution method: Molecular replacement in PHENIX with 3ILV as a starting model for hsNadE, and 3DLA for tbNadE-SFI; Composite omit map: CNS, PHENIX; 3D structure generator: CORINA; Ligand dictionary: XPLO2D; Structural validation: Molprobity, Quality Control Check from JCSG; Molecular tunnel analysis: CAVER; Steady-state rate constants; Prism 5 in GraphPad Software.

For manuscripts utilizing custom algorithms or software that are central to the research but not yet described in published literature, software must be made available to editors/reviewers. We strongly encourage code deposition in a community repository (e.g. GitHub). See the Nature Research guidelines for submitting code & software for further information.

Data

Policy information about availability of data

All manuscripts must include a data availability statement. This statement should provide the following information, where applicable:

- Accession codes, unique identifiers, or web links for publicly available datasets
- A list of figures that have associated raw data
- A description of any restrictions on data availability

The protein coordinates and structure factors of hsNadE in complex with NaAD+, AMP, and MgPPi, and tbNadE in complex with glutamine, SFI, and PPi have been deposited with PDB code 6OFB and 6OFC, respectively. Coordinate release: Hold for publication. Sequence release: Hold for release.

Field-specific reporting

Please select the one below that is the best fit for your research. If you are not sure, read the appropriate sections before making your selection.

☒ Life sciences ☐ Behavioural & social sciences ☐ Ecological, evolutionary & environmental sciences

For a reference copy of the document with all sections, see nature.com/documents/nr-reporting-summary-flat.pdf

Life sciences study design

All studies must disclose on these points even when the disclosure is negative.

Sample size	hsNadE complex: two data sets of low and high resolution containing 300 images were collected from a single crystal. tbNadE complex: 772 images were collected from a single crystal.
Data exclusions	X-ray diffraction used for structure determination of hsNadE complex was cut off at 2.84 angstrom resolution with Rsym of 58.6%, Rmerged of 118.3%, I/sI of 1.3, and redundancy of 4.2. For tbNadE complex , X-ray data was cut off at 3.14 angstrom resolution with Rsym of 42.7%, Rmerged of 64.4%, I/sI of 3.5, and redundancy of 8.7.
Replication	Table 1 and 3 data were performed in triplicate. Table 2 data were in replicates.
Randomization	*Describe how samples/organisms/participants were allocated into experimental groups. If allocation was not random, describe how covariates were controlled OR if this is not relevant to your study, explain why.*
Blinding	*Describe whether the investigators were blinded to group allocation during data collection and/or analysis. If blinding was not possible, describe why OR explain why blinding was not relevant to your study.*

Reporting for specific materials, systems and methods

We require information from authors about some types of materials, experimental systems and methods used in many studies. Here, indicate whether each material, system or method listed is relevant to your study. If you are not sure if a list item applies to your research, read the appropriate section before selecting a response.

Materials & experimental systems

n/a	Involved in the study	
☒	☐	Antibodies
☐	☒	Eukaryotic cell lines
☒	☐	Palaeontology
☒	☐	Animals and other organisms
☒	☐	Human research participants
☒	☐	Clinical data

Methods

n/a	Involved in the study	
☒	☐	ChIP-seq
☒	☐	Flow cytometry
☒	☐	MRI-based neuroimaging

Eukaryotic cell lines

Policy information about cell lines

Cell line source(s)	Sf9 insect cell lines: Invitrogen.
Authentication	Morphology check by microscope.
Mycoplasma contamination	Cell lines were purchased from company and were not tested for mycoplasma contamination.
Commonly misidentified lines (See ICLAC register)	*Name any commonly misidentified cell lines used in the study and provide a rationale for their use.*
